# A 3-year wastewater-based epidemiology study of noroviruses in Senegal

**DOI:** 10.1128/aem.01255-26

**Published:** 2026-07-27

**Authors:** Ngoné Diaw, Gérard Landry Boussiengui, Ndack Ndiaye, Mamadou Aliou Barry, Ousmane Kébé, Ndongo Dia, Ousmane Faye, Martin Faye

**Affiliations:** 1 Virology Department, Institut Pasteur de Dakar 89248, Dakar, Senegal; 2 Public Health Department, Institut Pasteur de Dakar 89248, Dakar, Senegal; Colorado School of Mines, Golden, Colorado, USA

**Keywords:** norovirus, wastewater environmental surveillance, epidemiology, statistical models, Senegal

## Abstract

Norovirus is a leading cause of acute gastroenteritis worldwide due to its high contagiousness, widespread transmission, and the lack of treatment or vaccine. In Senegal, no routine community-level monitoring is performed, and most available data concern other enteric viruses, leaving a gap in understanding norovirus circulation. To assess the potential of wastewater surveillance as an early warning system, we analyzed the spatiotemporal circulation in Senegal of noroviruses using clinical and environmental data collected between 2020 and 2023, combining statistical and machine learning models, and comparing effective reproduction number R(t) estimates between genotypes and surveillance systems. Our data show that norovirus GII was the dominant genotype during the study period. In addition, a strong spatial heterogeneity in viral load was identified between wastewater collection sites, with high titers observed in several wastewater collection sites. Interestingly, peaks of viral load in environmental setting frequently preceded increases in reported clinical cases. Modeling data indicated more persistent circulation of norovirus GII than GI. Our findings also highlighted the influence of temporal, demographic, and clinical factors for viral loads in wastewater. Comparison of the effective reproduction number R(t) confirmed the descriptive data and highlighted a more persistent circulation for norovirus GII and a detection of R(t)’s increases in wastewater data prior to variation in clinical data, underscoring the usefulness of wastewater environmental surveillance as an early warning tool system for norovirus surveillance. Our study is noteworthy by representing the first countrywide application of wastewater-based epidemiology to monitor norovirus circulation across diverse geographic and demographic settings in Senegal.

**IMPORTANCE:**

Noroviruses represent a major public health concern worldwide, particularly in low- and middle-income countries, such as Senegal where resources are limited. Monitoring noroviruses in wastewater is critical for early detection of outbreaks and tracking viral evolution, as these viruses are often underdiagnosed in clinical settings. In this study, we report on the spatiotemporal circulation of noroviruses in Senegal using clinical and environmental data collected over 4 years and combining modeling methods with estimation of epidemiological parameters. Our study provides the first countrywide data on the application of wastewater-based epidemiology to monitor noroviruses across diverse geographic and demographic settings in Senegal. Our data offer a proactive, cost-effective method to monitor public health trends and identify the presence of noroviruses within the population, offering an early warning system for community spread. The approach could be useful for the monitoring of other public health concerns in Senegal in the absence of routine clinical surveillance.

## INTRODUCTION

Viral gastroenteritis represents a major public health problem worldwide, particularly in low- and middle-income countries (1). Among the causative agents, noroviruses are the leading cause of both sporadic infections and large-scale epidemics (2). They cause millions of cases of diarrhea each year, affecting all age groups, with increased severity in children under 5 years of age, the elderly, and immunocompromised individuals (3). Transmission occurs primarily via the fecal-oral route through contaminated water, food, or infected surfaces. Despite their significant public health impact, no specific vaccine or antiviral treatment is currently available (4).

In many countries, particularly in Sub-Saharan Africa (SSA) where resources are limited, there is no specific norovirus surveillance system, which limits the ability to anticipate epidemics and guide public health interventions (5). In Senegal, available data mainly concern other enteric pathogens, such as rotaviruses (6), leaving a significant gap in knowledge regarding norovirus circulation dynamics.

Wastewater-based epidemiology (WBE) has proven its usefulness for the investigation of the relationship between the health condition of the population served and the concentrations of particular pathogens or medications in wastewater (7–9). The monitoring of a particular infectious illness over time is traditionally done through sentinel surveillance, the analysis of hospital admissions, the application of epidemiological surveys, and the calculation of morbidity and fatality rates. However, despite being valuable sources of information, these approaches require skilled workers for data collection and processing. In addition, this type of surveillance differs depending on the quality of information acquired because disease cases may go undetected by health services owing to misidentification or underreporting (9), as well as health-seeking behaviors and access to healthcare.

WBE offers a complementary approach to traditional methods and addresses some clinical surveillance limitations, providing fast and reliable information (10). It is a highly sensitive and a well-established tool for monitoring viruses with high rates of asymptomatic infection and substantial shedding, such as severe acute respiratory syndrome coronavirus 2 (SARS-CoV-2) and poliovirus (11–13). In addition, its potential application to norovirus monitoring has been already explored (14), offering a unique opportunity for non-invasive community-level surveillance.

To investigate the usefulness of environmental surveillance as an early warning tool for norovirus circulation within the community, we combined clinical and environmental data on noroviruses over 4 years in Senegal for a better understanding of their circulation dynamics using modeling and assessed the potential factors influencing their transmission to provide new insights into their epidemiology that could guide the existing surveillance strategies for acute viral gastroenteritis.

## MATERIALS AND METHODS

### Data collection

For this study, three datasets were used. The first data set was provided by the Sentinel Syndromic Surveillance in Senegal network (4S network) (15). It included cases of acute and febrile diarrhea reported through the 4S network between January 2020 and December 2023. The data were aggregated over time and included the date of notification as well as the number of cases recorded. These syndromic indicators were used as indirect measures of enteric disease activity in the population. However, they should not be interpreted as laboratory-confirmed norovirus infections, as they include only notifications.

The second data set consisted of laboratory-confirmed norovirus infections identified in stool samples from patients presenting with acute gastroenteritis through an agile surveillance conducted in the framework of the 4S network in Senegal between September 2020 and January 2021 (Fig. S1). Laboratory testing was performed at the National Reference Center (NRC) for Poliomyelitis at Institut Pasteur de Dakar (IPD).

The third data set was generated through a surveillance program of noroviruses in wastewater samples collected between January 2020 and December 2023 from 15 sites, including 12 wastewater treatment plants (WWTPs) and three open canals across seven regions of Senegal, conducted by the National Reference Center for Poliomyelitis at IPD. Available variables included the sample’s collection date, the sampling site’s geolocation and name, the name of the geographical region, the lab data on norovirus detection, and viral load’s quantification data.

### Sample collection and laboratory processing

#### Wastewater samples

A total of 580 wastewater samples were collected between January 2020 and December 2023 from 15 sampling sites across seven regions of Senegal. Samples were collected upstream of any wastewater treatment and then transported and stored according to World Health Organization (WHO) standardized procedures. Viral particles were concentrated using the polyethylene glycol/dextran (PEG/Dextran) biphasic method, followed by viral RNA extraction using the Zymo RNA Extraction Kit according to the fabricant’s instructions. Norovirus detection and quantification were performed by real-time RT-PCR. Results were expressed as cycle threshold (Ct) values, and the viral load in positive samples was calculated using a standard equation.

#### Stool samples

Stool samples were 10% diluted in 1× phosphate-buffered saline (PBS). Viral RNA was extracted from the stool suspensions using the Zymo RNA Extraction Kit according to the fabricant’s instructions. RNA extracts were tested using a specific multiplex real-time RT-PCR assay for the detection of norovirus, rotavirus, and adenovirus infections. The available variables included patient demographics, sampling date, laboratory results for norovirus, rotavirus, and adenovirus, the presence of any viral co-infections, and the main clinical symptoms reported.

### Statistical data analysis

A univariate analysis was performed to describe the study population and the main characteristics of community-acquired infections. Associations between norovirus positivity and explanatory variables were explored using the chi-square test or Fisher’s exact test for categorical variables.

Concurrently, a descriptive analysis was conducted to describe the epidemiology of norovirus infections based on clinical and environmental data. The results were presented graphically to illustrate the temporal dynamics of infections, their geographic distribution, and the evolution of viral load in wastewater and clinical samples.

### Statistical modeling

The generalized linear model (GLM) with a gamma distribution was used to assess the influence of temporal, demographic, and environmental factors on viral loads in wastewater. The gamma distribution was adapted to the positive and skewed nature of the data (16), with reference values based on the lowest median. Multinomial regression identified factors associated with the detection of NoVGI and NoVGII, while binary logistic regression was used to identify factors independently associated with rotavirus, adenovirus, and norovirus infections in the clinical population. Furthermore, a weighted random forest was applied to correct class imbalance and improve the prediction of viral infections (17), and an epidemiological approach to wastewater explored the early detection and monitoring of community viral circulation. All analyses were performed using RStudio (version 4.1.3), ensuring consistency and reproducibility.

## RESULTS

### Exploratory and descriptive analysis

#### Clinical monitoring

Of the 424 cases of gastroenteritis recorded between September 2020 and January 2021, 59 (13.9%) were positive for norovirus. Among these positive cases, 54 (91.5%) were identified as being due to genotype II norovirus (NoVGII) and 5 (8.5%) as being due to genotype I norovirus (NoVGI), indicating a predominance of NoVGII. No significant association was observed with demographic, contextual, or clinical factors. However, the year of sample collection was significantly associated with norovirus detection (*P* = 0.005), with a higher proportion in 2021 than in 2020. A significant difference was also observed according to the sampling center (*P* = 0.040), suggesting geographical heterogeneity in viral circulation. No significant co-infection with rotavirus or adenovirus was observed (*P* > 0.05), although non-significant trends were noted for the presence of blood in the stool and headaches (Table S1).

**Fig 1:**
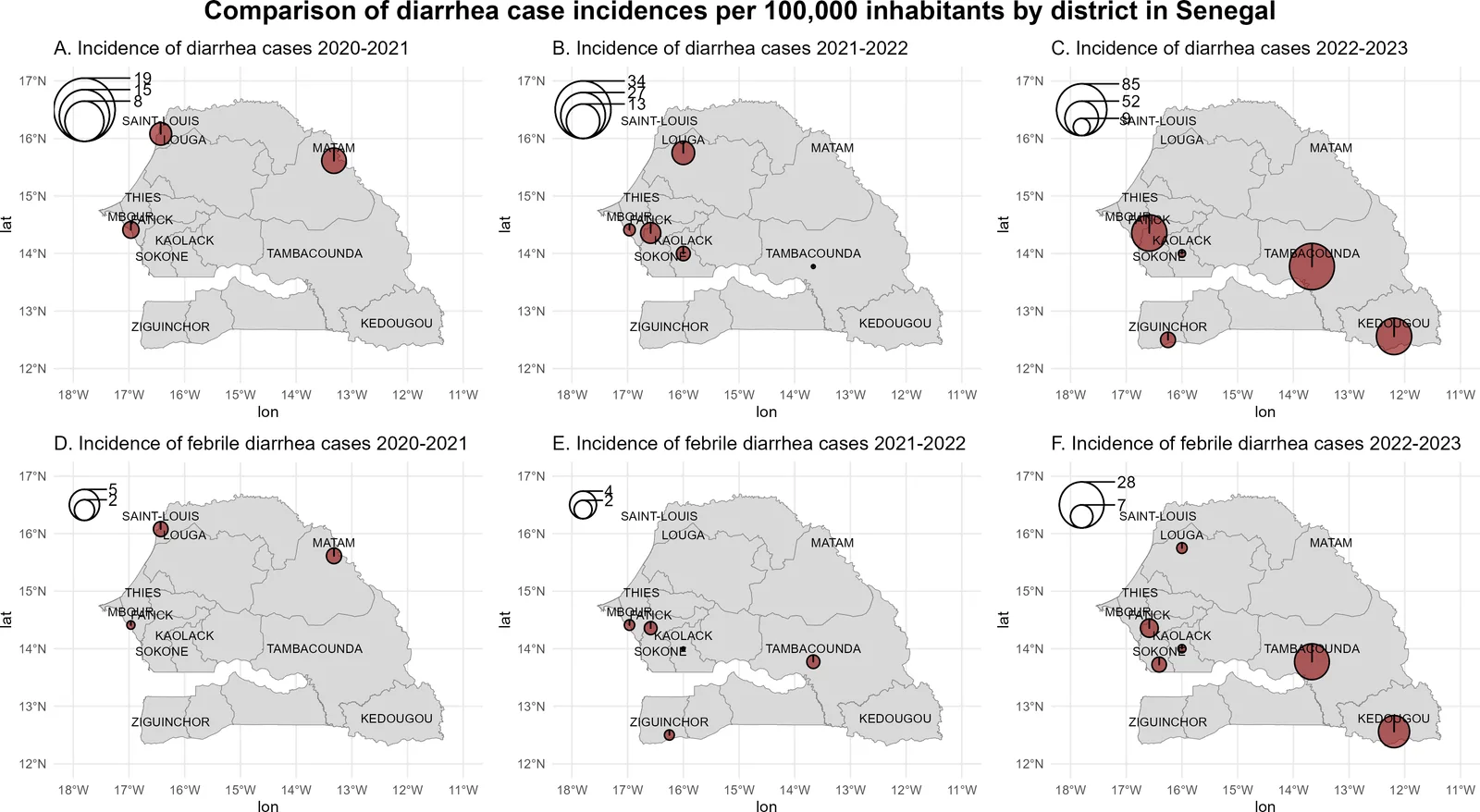
Maps of the incidence of reported cases of diarrhea in clinical data between 2020 and 2023. The annual incidence is represented by circles in red positioned by district, the size of which is proportional to the observed incidence. The nested legend indicates the scale of values.

The spatial analysis of diarrhea incidence between 2020 and 2023 reveals marked geographical heterogeneity among districts, with the most affected areas varying from year to year and a progressive increase in incidence in several districts in the east and center of the country. Overall, a rise in diarrhea cases was observed between 2020 and 2023, particularly in Tambacounda, Kédougou, Fatick, and Ziguinchor, suggesting the existence of persistent transmission foci, while the incidence of febrile diarrhea remained generally low despite a notable increase in 2022–2023 in the districts of Tambacounda and Kédougou (Fig. 1).

The incidence of reported diarrheal cases in the districts of the Dakar region remained generally low throughout the study period, with slight variations between districts during the years 2020–2022 (Fig. S2). No incidence was reported between 2022 and 2023, although possible underreporting of mild cases cannot be ruled out.

#### Viral prevalence in clinical samples

The most frequently reported symptoms in patients infected with NoVGII were diarrhea (12%, *n* = 54), vomiting (2.59%, *n* = 11), and abdominal pain (1.56%, *n* = 7), consistent with the literature (18), while other symptoms, such as fever, rhinorrhea, and headache, were observed less frequently (Fig. S3). No clinical symptoms were specifically associated with norovirus infection.

Analysis of patient management shows a predominance of the oral rehydration salts (ORS) + zinc combination prescribed in 78% of cases (*n* = 348), regardless of virologic status. Among the patients, 12% were positive for NoVGII (*n* = 52), while 79% were negative (*n* = 335), confirming that this combination is the standard treatment for acute diarrhea.

Other treatments (antispasmodics, antibiotics, antiparasitics, antipyretics, antimalarials, antitussives, and antiemetics) are rarely used. The majority of patients are placed under observation (9.66% of negative cases, *n* = 41; 1.41% of NoVGII-positive cases, *n* = 6), while hospitalization (1.17%, *n* = 5) and referrals (0.47%, *n* = 2) remain exceptional (Fig. S4).

**Fig 2:**
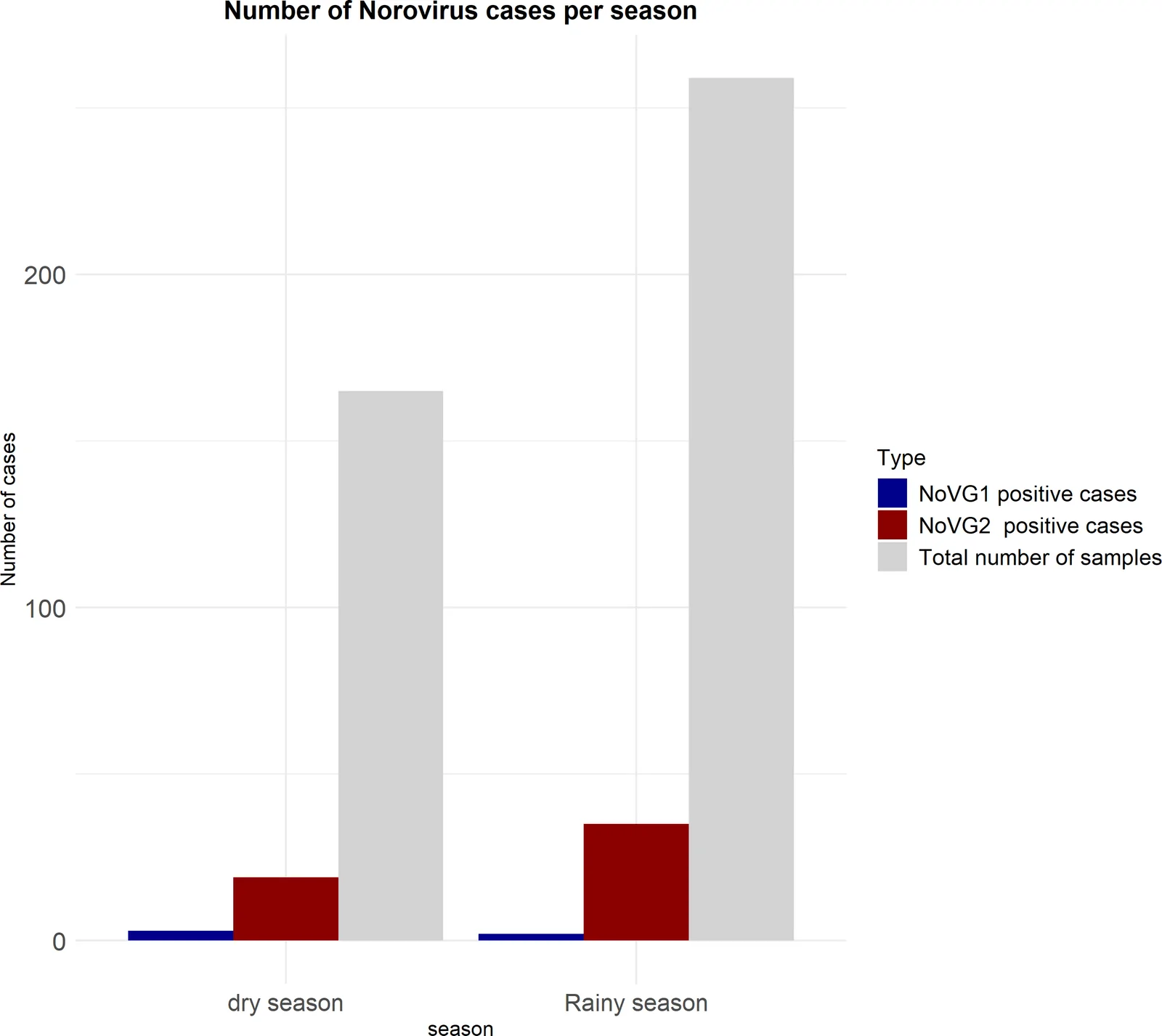
Number of confirmed norovirus cases detected in clinical samples by season between 2020 and 2021. The bar chart shows NoVGI (red), NoVGII (blue) cases, and the total number of samples analyzed (gray) for each season.

In Senegal, the rainy season extends from August to October and the dry season from November to July. Samples, as well as positive cases of NoVGII, were more frequent during the rainy season, while cases of NoVGI, although fewer in number, appeared slightly more frequent during the dry season (Fig. 2).

Analysis of clinical results reveals a clear predominance of NoVGII detected in the majority of districts, with a particularly high prevalence in Tambacounda (28.3%), Guédiawaye (21.9%), and Saint-Louis (18.3%). NoVGI appears less common, with a prevalence generally below 5%, except in the Dakar South district where it reaches 12.5%. Adenoviruses are mainly observed in Kaolack (33.3%) and Tambacounda (13.0%), while rotaviruses are concentrated in Matam (14.3%) and Sokone (10.0%) (Fig. S5).

#### Coinfections in clinical samples

**Fig 3:**
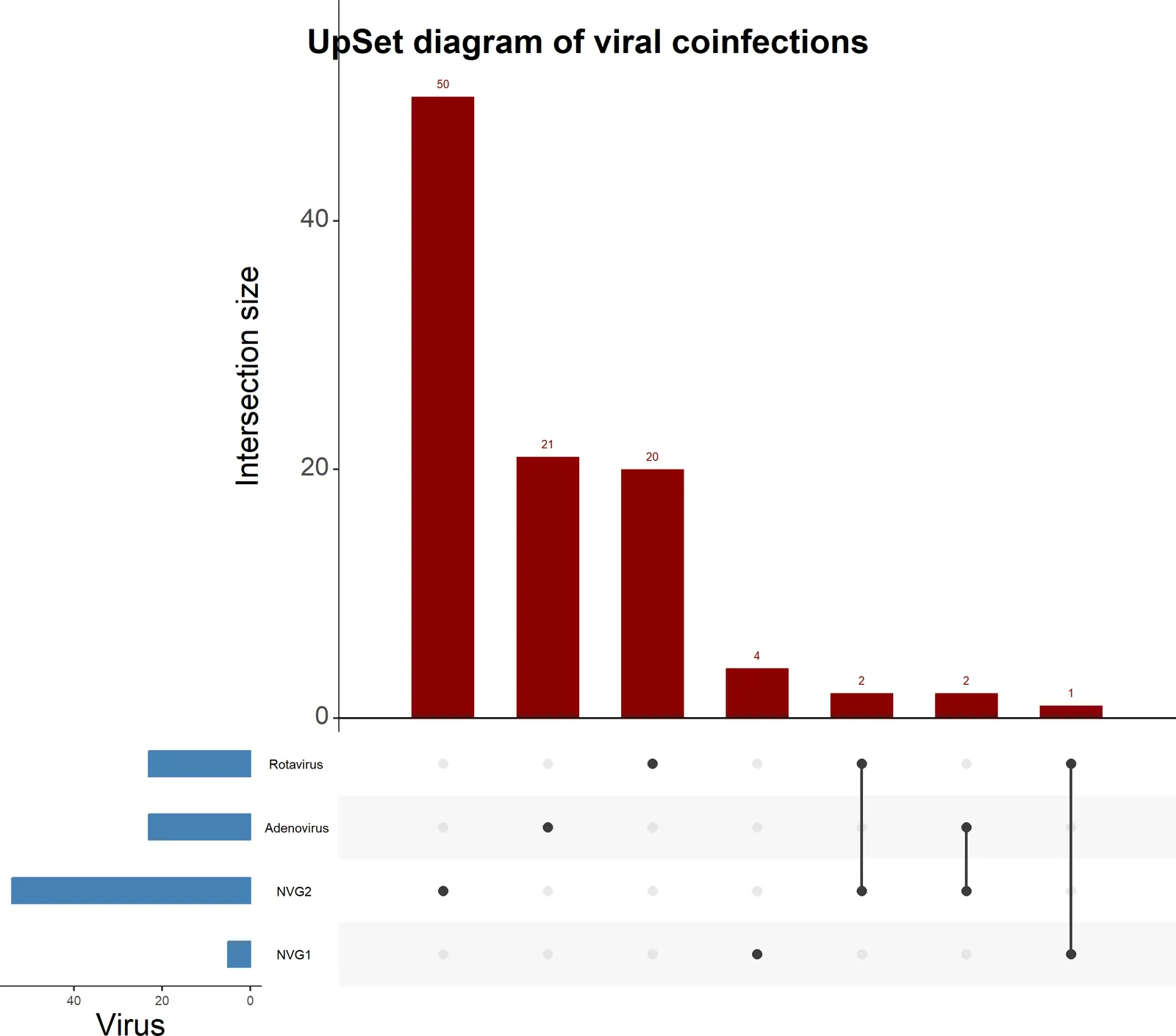
UpSet plot of viral co-infections detected in clinical samples collected between 2020 and 2021. The vertical bars (red) represent the number of cases corresponding to each combination of infections. The horizontal bars (blue) indicate the total number of positive cases for each virus. The black dots connected by vertical lines identify the virus combinations OIK UQA observed in cases of co-infection.

Analysis of viral co-infections revealed limited but significant associations. The results showed co-infections between NoVGII and rotavirus (two cases), between NoVGII and adenovirus (two cases), and between NoVGI and rotavirus (one case). However, no simultaneous co-infections between NoVGI and NoVGII were observed in the clinical database (Fig. 3).

#### Wastewater monitoring

**Fig 4:**
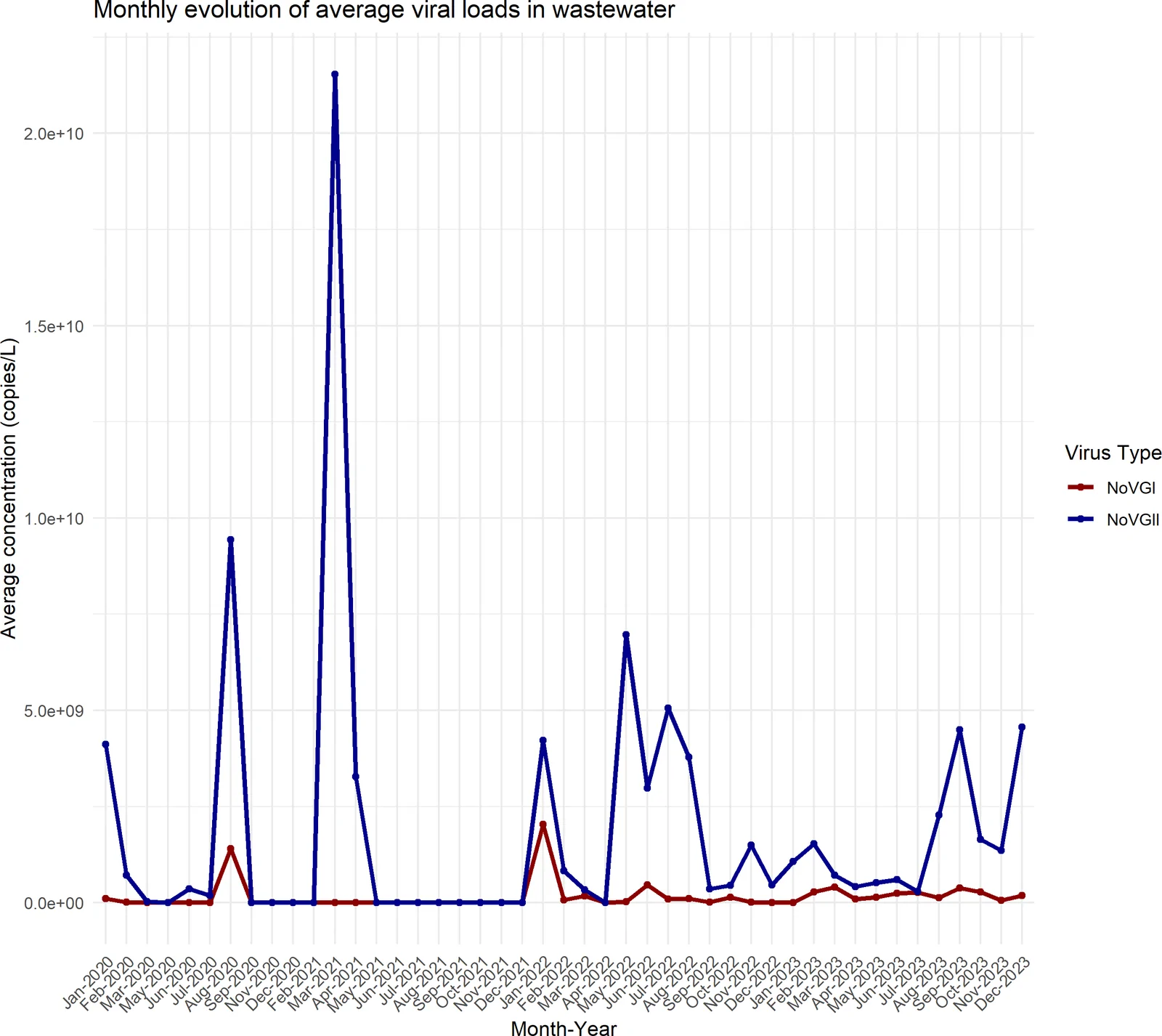
Monthly distribution of norovirus viral loads in environmental samples collected between 2020 and 2023. The curves represent the average monthly viral loads of NoVGII (blue) and NoVGI (red).

Viral loads for NoVGII show marked peaks, particularly in August 2020, March 2021, January 2022, May to August 2022, July to October 2023, and December 2023, with concentrations exceeding 2.0 × 10^10^ copies/L for the highest peak in March 2021. NoVGII is largely dominant throughout the study period, confirming its leading role. Conversely, NoVGI exhibits lower viral loads, with limited fluctuations and occasional peaks, although both genogroups follow a similar temporal trend (Fig. 4).

Analysis of environmental data from wastewater treatment plants confirms the predominance of NoVGII nationwide, with high prevalence rates at several sites, while NoVGI is generally detected at lower levels, with a few sites showing higher proportions (Fig. S6). In the Dakar region, the “Cambérène” and “Khourounar” sites characterized by a large number of samples show high circulation of NoVGII (43.7 and 47.1%, respectively) associated with significant co-circulation of NoVGI (18.7 and 25.7%). A variable proportion of negative samples is observed across sites, suggesting spatial heterogeneity in environmental viral circulation.

#### Comparative analysis of circulation trends

The period between weeks 35 and 44 corresponds to the hot rainy season in Senegal (August–October) characterized by high rainfall and humidity. Weeks 45 to 44 correspond to the cool dry season (November–January) marked by lower temperatures and no rain, while weeks 5 to 34 correspond primarily to the hot dry season (February–July) characterized by high temperatures and minimal rainfall.

**Fig 5:**
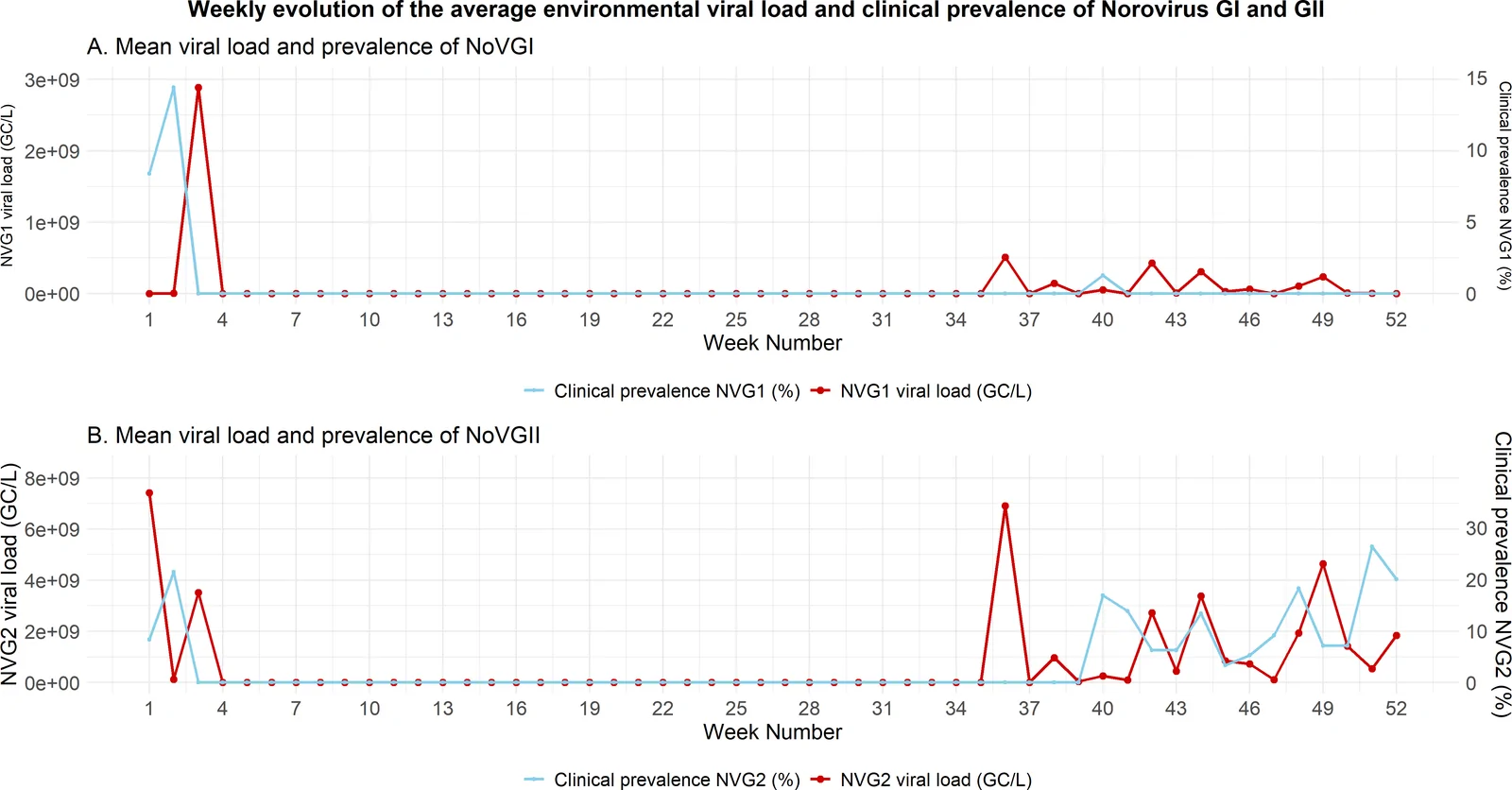
Line graphs show NoVGI (A) and NoVGII (B) wastewater viral loads peaking near weeks 1 to 4 and again around weeks 35 to 52, with clinical prevalence following similar seasonal patterns.

The comparative analysis of trends from clinical and environmental data reveals a difference in behavior between norovirus genotypes depending on the season. Genotype GII shows a strong correlation between viral loads measured in the environment and clinical prevalence, with a joint increase in viral loads in wastewater and clinical cases observed mainly at the transition between the rainy and cool dry seasons. Similarly, the GI genotype circulates widely in the environment, sometimes with high viral loads, particularly during the rainy season, without a proportional increase in clinical cases (Fig. 5).

### Statistical modeling

#### Regression models on clinical data between 2020 and 2021

The results from the multivariate analyses comparing the different viral infections are summarized in Table S2. Given the number of models estimated, only the significantly associated factors (*P* < 0.05), as well as the main goodness-of-fit and discriminant performance indicators, are presented.

Multinomial regression reveals distinct profiles according to the norovirus genotype. NoVGI is associated with the year of sample collection, rotavirus coinfection, certain clinical symptoms (rhinorrhea, blood in the stool), and the area of residence, while NoVGII is primarily linked to the year of sample collection, myalgia, and the area of residence.

Binary logistic regression models confirm the importance of the year of sample collection and the area of residence as common determinants of norovirus, adenovirus, and rotavirus infections. The AUC values lower than those of the multinomial model,range from 0.68 for norovirus to 0.86 for rotavirus, indicating low to moderate predictive power. The pseudo-*R*² values, although modest, reflect the multifactorial complexity of the data and suggest the involvement of unobserved factors (Table S2).

#### Comparison of discriminating factors according to infection using random forest

Weighted random forest analysis identified the main discriminating variables within the overall population and specific profiles for norovirus, adenovirus, and rotavirus infections.

In the study population, the most important variables reflect contextual and clinical factors, including the year and season of sample collection, area of residence, and gastrointestinal and extra-gastrointestinal symptoms (vomiting, blood in the stool, cough, and rhinorrhea).

Targeted virus-specific analysis reveals distinct profiles. For norovirus, the discriminating variables primarily include extra-gastrointestinal symptoms and social factors, with headache, area of residence, cough, and abdominal pain, participation in gatherings, sex, and vomiting as the main determinants. Adenovirus infections are more strongly associated with age, cough, vomiting, year of sample collection, and area of residence. Rotavirus infection is primarily characterized by season, sex, age, vomiting, and place of residence.

**Fig 6:**
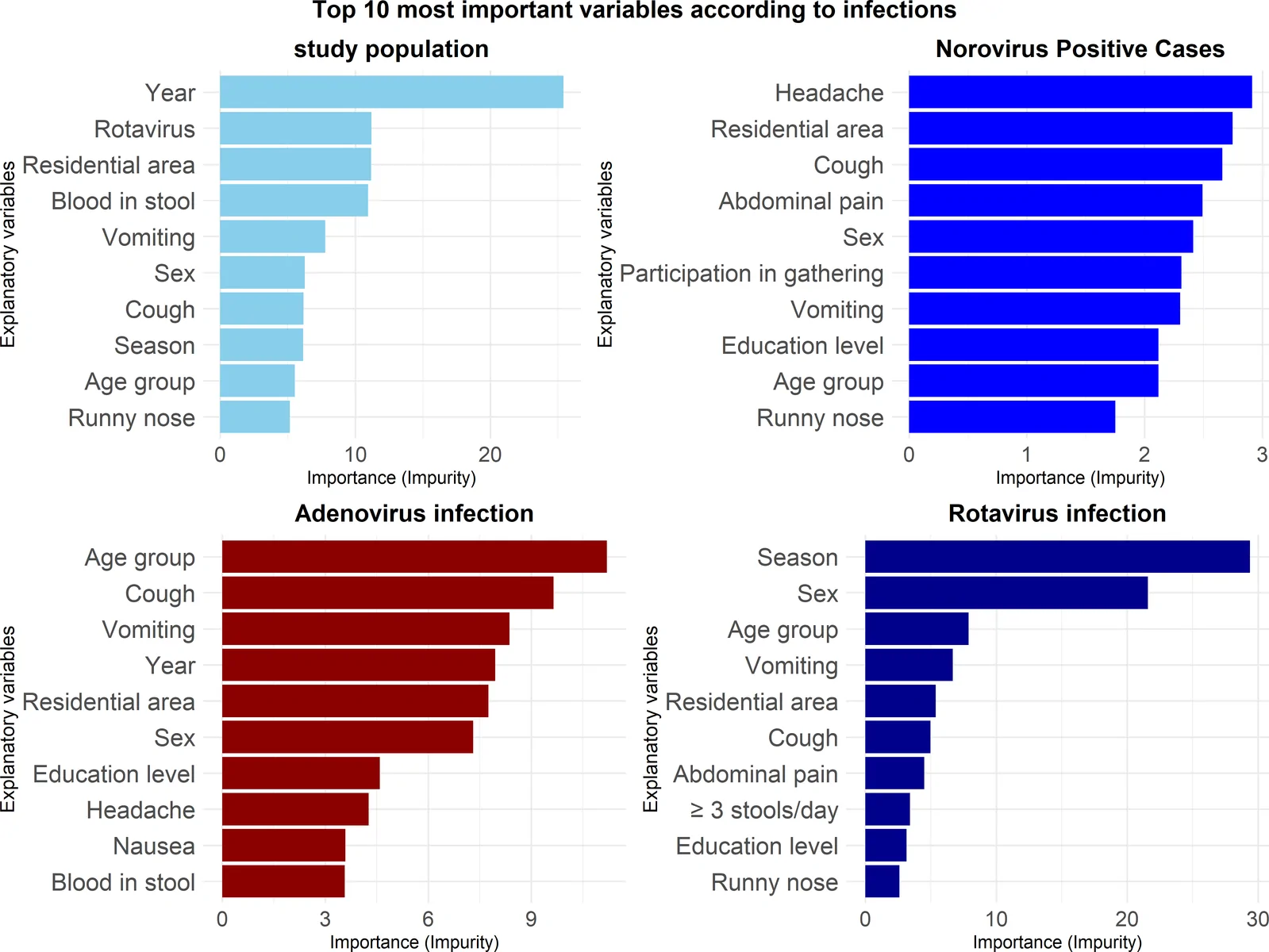
Importance of variables according to the study population and viral infections using weighted random forest. The colors indicate the modeling of the study population (light blue, multinomial regression) and of adenovirus (red), rotavirus (dark blue), and norovirus (blue) infections by binary logistic regression.

Comparison of clinical profiles shows that norovirus and adenovirus share some determinants (cough, vomiting, place of residence) but differ: norovirus is influenced by social and contextual factors, while adenovirus is more closely linked to age and gastrointestinal symptoms. Similarly, rotavirus is distinguished by its seasonality, whereas norovirus combines social and symptomatic factors. Overall, the norovirus profile is more heterogeneous, incorporating contextual, social, and clinical dimensions (Fig. 6).

Comparing binary logistic regression and weighted random forests highlights their complementarity in analyzing the determinants of norovirus infections. Logistic regression identifies clear and interpretable directional associations through odds ratios (OR), while random forests confirm the importance of these variables by revealing complex and non-linear interactions between contextual, sociodemographic, and symptomatic factors. Thus, logistic regression stands out for its explanatory power, while random forests are characterized by their predictive power. Their combined use provides a more comprehensive understanding of the dynamics of norovirus infections (Table S1; Fig. 6).

#### Modeling the average viral load for NoVGI and NoVGII using gamma MLG

##### NoVGII

For norovirus GII, the month of September, the “Korentas site,” and the rainy season were defined as reference categories in the model.

The month of September, the “Korentas site,” and the rainy season were chosen as reference categories. The generalized linear gamma distribution model applied to NoVGII viral loads highlights strong spatial heterogeneity. With the exception of the “Keur Niang station,” all stations show concentrations higher than the reference site, with particularly high levels at “Khourounar” (×55), “Tivaouane Peulh” (×52), “SHS Cité Enseignant” (×46), and “Cap des Biches” (×35). No significant differences were observed between seasons, although some months, notably August, suggest a moderate temporal variation (Table S3).

The model explains 20.8% of the variability in viral loads and exhibits moderate overdispersion (2.49), reflecting the heterogeneity of the environmental data.

##### NoVGI

For norovirus GI, the month of January, the “Kaolack pumping station,” and the dry season were defined as reference categories in the model. The generalized linear model applied to NoVGI viral loads revealed significant variations according to the sampling site and the month, with no significant effect of the season. The “Camberène,” “Ouakam,” “SHS Cité Enseignant,” and “Tivaouane Peulh” sites showed viral loads approximately five to seven times higher than those of the reference site. Temporally, only February was associated with significantly lower viral loads than the reference month; the other months showed non-significant or marginal variations (Table S4).

The gamma-distributed GLM applied to NoVGII viral loads explained 23.5% of the variability, with moderate overdispersion (2.27) reflecting the heterogeneity of the environmental data.

### Wastewater epidemiology and estimates of the reproduction rate R0(t)

#### LOESS smoothing and proxy incidence calculation

We observe variable temporal dynamics of NoVGI and NoVGII in wastewater marked by fluctuations suggesting successive epidemic waves, with greater circulation of NoVGII. The incidence indicator derived from viral loads generally follows the trends observed by locally estimated scatterplot smoothing (LOESS), confirming its relevance as an indirect indicator of viral circulation. This consistency justifies its use for estimating the effective reproduction number (Fig. S7).

#### Monthly evolution of proxy incidences

The monthly evolution of NoVGI incidence between 2020 and 2023 shows irregular circulation, with a marked increase starting in late 2021 and a major peak between November 2021 and January 2022. Subsequent fluctuations suggest sporadic epidemic episodes possibly linked to seasonal or environmental factors. Conversely, NoVGII exhibits more continuous circulation, with a gradual increase starting in 2021 and several epidemic peaks, including a major peak between May and June 2022. This persistent dynamic confirms the dominance of NoVGII capable of maintaining endemic circulation with successive waves (Fig. S8).

#### NoVGI transmission in wastewater

The proxy incidence of NoVGI shows strong temporal variability between 2020 and 2023, with low activity in 2020 and marked peaks in early 2022, followed by low to moderate circulation, suggesting short, seasonal transmission episodes. The serial interval is mostly between 2 and 5 days, indicating rapid secondary transmission. The estimate of the effective reproduction number R(t) shows fluctuations around the epidemic threshold, with values above 1 during periods of resurgence and mostly below 1 in 2023, reflecting moderate endemic circulation and progressive control of transmission (Fig. S9).

#### NoVGII transmission in wastewater

The proxy incidence of NoVGII shows low activity between 2020 and 2021, followed by extended peaks between February and August 2022, and then between August and December 2023, indicating more persistent and irregular transmission waves than for NoVGI. NoVGI and NoVGII share a similar serial interval, mostly between 2 and 5 days. The estimation of the effective reproduction number R(t) reveals prolonged periods with R(t) > 1, sometimes close to 2, reflecting sustained and continuous transmission of NoVGII, consistent with its role as the dominant genotype in human gastroenteritis (Fig. S10).

#### Comparative analysis of the reproduction rates from different types of diarrhea between 2020 and 2023

**Fig 7:**
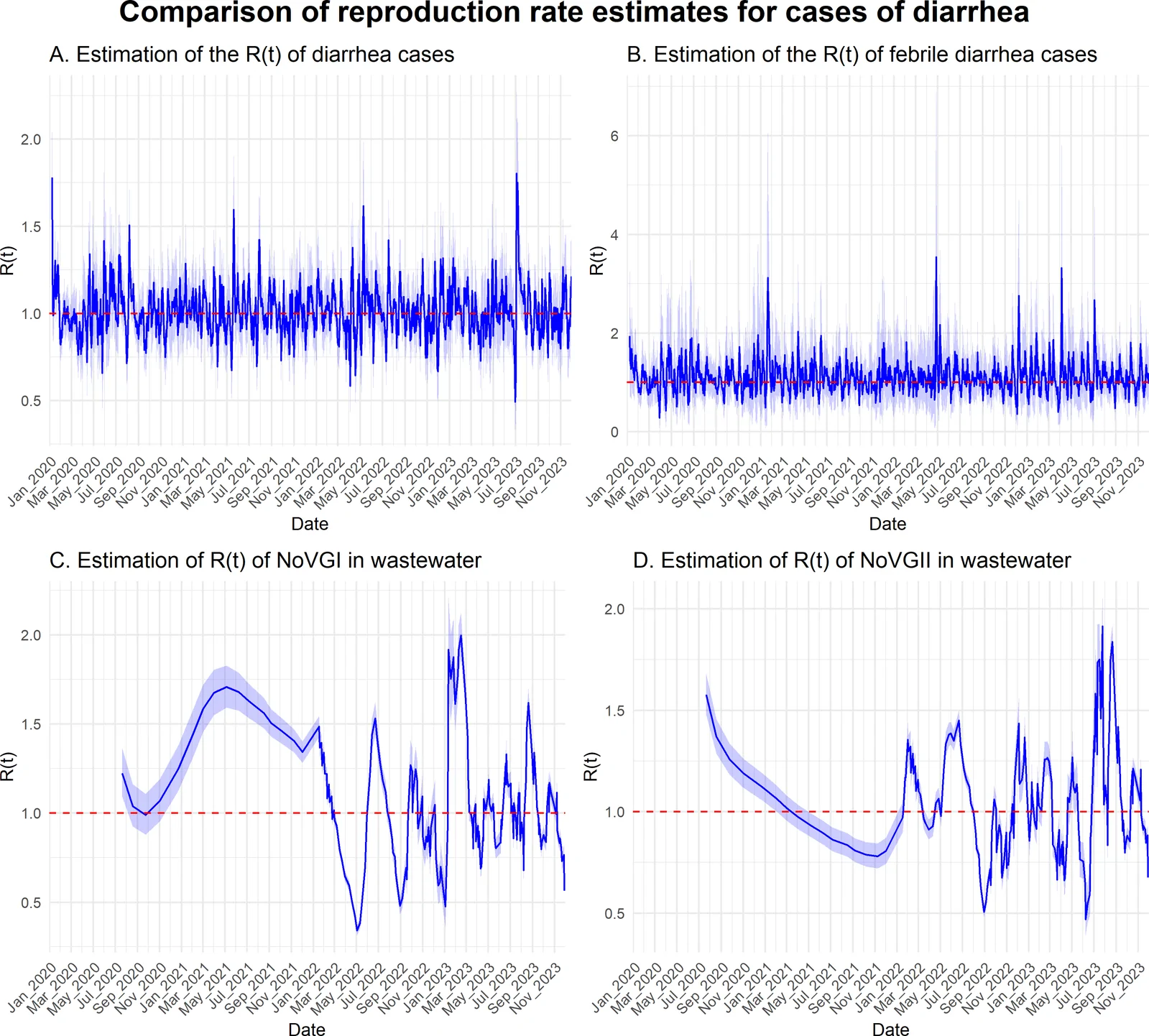
Comparison of the distribution of R(t) according to types of diarrhea between 2020 and 2023. (**A**) Confirmed cases of diarrhea; (**B**) confirmed cases of febrile diarrhea; (**C**) NoVGI infections estimated from wastewater; and (**D**) NoVGII infections estimated from wastewater.

Analysis of the reproduction number R(t) for diarrhea and febrile diarrhea shows an overall stable dynamic between 2020 and 2023, with fluctuations around the epidemic threshold of 1. The higher values observed for febrile diarrhea are linked to high variability due to the low number of cases. In contrast, NoVGI and especially NoVGII exhibit more pronounced dynamics in wastewater, with repeated periods where R(t) > 1, reflecting more intense and seasonal circulation, particularly for GII (Fig. 7).

The comparison shows that wastewater reflects or precedes community case peaks, suggesting that wastewater surveillance can serve as an early warning system for infection spikes in the community.

## DISCUSSION

Herein, a wastewater-based epidemiology approach has been applied to estimate human exposure to noroviruses in Senegal and assess the contribution of environmental surveillance as an early-warning tool for a better understanding of the viral circulation dynamics. Our descriptive data analyzing the presence of noroviruses in influents of 12 small municipal wastewater treatment plants (WWTPs) and three open canals handling domestic sewage, together with clinical and laboratory data from acute diarrheal disease collected through the 4S network in Senegal between 2020 and 2023, provide new insights into the epidemiology of noroviruses in Senegal.

The increase in diarrhea cases reported between 2020 and 2023 in some districts could be associated with the persistence of adverse environmental and structural factors, such as limited access to quality drinking water, insufficient sanitation infrastructure, and inadequate hygiene practices, promoting a lasting fecal hazard and the transmission of enteric agents (19, 20).

The predominance of oral rehydration therapy combined with zinc is consistent with current therapeutic recommendations for the management of acute gastroenteritis, which emphasize supportive care in the absence of a specific treatment for norovirus infections (21, 22).

The seasonal variations observed in Senegal suggest a probable influence of climatic factors on the transmission dynamics of norovirus as previously described (23). Similar trends have been previously reported in Cameroon, where the circulation of noroviruses, particularly the NoVGII, peaks at the beginning of the rainy season, suggesting the influence of environmental factors, such as humidity and rainfall on viral transmission (24). However, further investigations focusing on the comparison of our data with trends described in other African contexts are warranted. In addition, the increased survival capacity of noroviruses at low temperatures, especially the NoVGII, could contribute to the persistence of cases during the cooler periods of the year (25).

Although less frequent than single infections, the viral co-infection rates identified in our study are consistent with previous data describing the occurrence of co-infection in patients of all ages due to the simultaneous circulation of several enteric viruses, such as norovirus, rotavirus, and adenovirus, in highly contaminated environments (26). The predominance of NoVGII observed in several districts in our study confirms its widespread and persistent circulation worldwide and is consistent with previous data reported in Africa, while NoVGI generally remains less frequent (27, 28). Peaks identified in viral loads from environmental data associated with the overall increase in the prevalence of NoVGII suggest a high rate of norovirus circulation in the community. Similar patterns have been widely reported in environmental surveillance studies and are often attributed to seasonal, climatic, and behavioral dynamics (29, 30). Despite its detection at lower levels, NoVGI exhibited similar trends.

Statistical models applied in our study indicate that variations in the detection rates of noroviruses are associated with the year of sampling, the area of residence, co-infection with rotavirus, and particular clinical symptoms, such as the presence of blood in the stools and headaches. These associations confirm the existence of spatial and temporal variabilities in the circulation of noroviruses as previously described (31, 32). In addition, binary and multinomial regression models identified directional associations between several risk factors, although their predictive power remains limited, primarily due to class imbalance and the small number of discriminating variables. However, the weighted random forest model revealed significant positive associations between the viral load and the contextual, social, and clinical factors, underscoring the value of non-linear approaches for studying enteric infections.

Our data showed that age appears to be the main factor associated with adenovirus infections, reflecting increased vulnerability in young children, consistent with previous data (33–35). Vomiting and exposure to risky environments, particularly collective or insufficiently chlorinated environments, are factors associated with fecal-oral transmission of viral gastroenteritis, while coughing and sex are not direct factors of gastrointestinal infection due to adenoviruses, which makes their association difficult to interpret in our study, especially in the context of a small number of positive cases (33). However, we identified a primary association of rotavirus infections with seasonality, sex, and place of residence, confirming its strongly seasonal circulation trends and the differences in exposure between urban and rural environments (36, 37).

Environmental analysis reveals marked spatial heterogeneity in norovirus viral loads, with high concentrations observed at specific sites probably related to absolute humidity, socioeconomic index, population density, and sanitation system characteristics. Similar data have been reported in previous studies monitoring enteric viruses in wastewater in Europe (38) and Asia (39). Although being more moderate, the temporal variability in viral loads suggests an influence of climatic conditions on the persistence and detection of the virus (40).

The application of WBE, including R(t) estimation, allowed a more detailed exploration of the temporal dynamics of noroviruses in Senegal. R(t) values associated with NoVGII frequently exceed the epidemic threshold, indicating also a more sustained transmission and a greater persistence compared to NoVGI. These findings confirm previous data describing NoVGII as the most prevalent norovirus genotype characterized by high environmental stability and implicated in the majority of recurrent epidemics (41–43).

The combination of environmental monitoring and R(t) estimates suggests a sustained circulation of NoVGII, which was frequently detected above the epidemic threshold during our study period. In addition, the temporal consistency between environmental signals and clinical data, sometimes with prior detection in environmental settings, underscores the potential of wastewater-based surveillance to serve as an early warning tool and a complementary approach supporting public health (29). However, non-confirmed and febrile diarrhea clinical data should be interpreted cautiously, as these syndromic indicators may reflect infections caused by multiple enteric pathogens and are not specific to norovirus infection. This approach is in line with the strategy of early warning systems based on continuous monitoring and analysis of environmental data, making it possible to anticipate health threats, such as noroviruses, and strengthen surveillance systems by moving from reactive to proactive management (44).

Our study is noteworthy for representing the first nationwide application of wastewater-based epidemiology to monitor both NoVGI and NoVGII circulations across diverse geographic and demographic settings in Senegal. We identified a sustained circulation of noroviruses in Senegal characterized by the predominance of NoVGII and heterogeneous spatiotemporal dynamics. The integration of clinical and environmental data, combined with R(t) estimation, provides new insights into community transmission and emphasizes the value of wastewater-based surveillance as a strategic tool for monitoring and outbreak preparedness. These findings underscore the need to promote integrated surveillance systems to improve the response to enteric infections in the African context.

Nevertheless, our study, has certain limitations that must be considered when interpreting the results. The relatively small number of samples in some districts and the imbalance between classes may have reduced the statistical power of the models and their predictive capacity. Furthermore, data on some clinical and contextual factors s that could influence transmission were not available, which may introduce residual confounding bias. Finally, wastewater-based surveillance, while informative, does not allow for the direct identification of infected individuals or for an accurate estimation of the true incidence at the community level.

## Data Availability

All data are included in the published article. The data sets used in the current study are available from the corresponding author and will be made available on request.

## SUPPLEMENTAL MATERIAL

Supplemental material aem.01255-26-s0001.pdf Tables S1 to S4; Fig. S1 to S10.

ASM does not own the copyrights to Supplemental Material that may be linked to, or accessed through, an article. The authors have granted ASM a non-exclusive, world-wide license to publish the Supplemental Material files. Please contact the corresponding author directly for reuse.
