# Supplementary material for: A 3-year wastewater-based epidemiology study of noroviruses in Senegal
Source: Appl Environ Microbiol. 2026 Jul 27;92(8):e01255-26. doi: 10.1128/aem.01255-26 (PMC13488322; doi:10.1128/aem.01255-26)
Supplement: Supplementary file 1 [file aem.01255-26-s0001.pdf]

Title: A three-year wastewater-based epidemiology study of noroviruses in Senegal.

A. Supplementary Tables

Table S1: Characteristics of norovirus gastroenteritis cases between 2020 and 2021.

Characteristics	Norovirus not detected n = 365	NoVGI detected n = 5	NoVGII detected n = 54	Total n = 424	p-value
Demographic factors					
Sex					>0.9[1]
Women	86.3% [246]	1.1% [3]	12.6% [36]	100.0% [285]	
Men	85.6% [119]	1.4% [2]	12.9% [18]	100.0% [139]	
Age group					>0.9[1]
0-5	85.2% [299]	1.4% [5]	13.4% [47]	100.0% [351]	
19-34	88.5% [23]	0.0% [0]	11.5% [3]	100.0% [26]	
35-59	93.8% [15]	0.0% [0]	6.3% [1]	100.0% [16]	
6-18	87.0% [20]	0.0% [0]	13.0% [3]	100.0% [23]	
60+	100.0% [8]	0.0% [0]	0.0% [0]	100.0% [8]	
Level of Study					0.6[1]
None	85.5% [318]	1.3% [5]	13.2% [49]	100.0% [372]	
Quranic school	100.0% [7]	0.0% [0]	0.0% [0]	100.0% [7]	
Primary	82.8% [24]	0.0% [0]	17.2% [5]	100.0% [29]	
Secondary or higher education	100.0% [16]	0.0% [0]	0.0% [0]	100.0% [16]	
Occupation					0.6[1]
Other profession	100.0% [6]	0.0% [0]	0.0% [0]	100.0% [6]	
Shopkeeper	50.0% [1]	0.0% [0]	50.0% [1]	100.0% [2]	
Pupil/Student	76.9% [10]	0.0% [0]	23.1% [3]	100.0% [13]	
Employee	100.0% [1]	0.0% [0]	0.0% [0]	100.0% [1]	
Housewife	94.4% [17]	0.0% [0]	5.6% [1]	100.0% [18]	
Worker	100.0% [2]	0.0% [0]	0.0% [0]	100.0% [2]	

**Characteristics**	**Norovirus not detected** n = 365	**NoVGI detected** n = 5	**NoVGII detected** n = 54	**Total** n = 424	**p-value**
Unemployed	85.9% [328]	1.3% [5]	12.8% [49]	100.0% [382]	
Contextual factors					
Pregnant woman					0.5¹
No	86.2% [361]	1.2% [5]	12.6% [53]	100.0% [419]	
Yes	80.0% [4]	0.0% [0]	20.0% [1]	100.0% [5]	
Participation in a gathering					0.11¹
No	85.3% [342]	1.2% [5]	13.5% [54]	100.0% [401]	
Yes	100.0% [23]	0.0% [0]	0.0% [0]	100.0% [23]	
Surrounded by cases of diarrhea					>0.9¹
No	85.9% [348]	1.2% [5]	12.8% [52]	100.0% [405]	
Yes	89.5% [17]	0.0% [0]	10.5% [2]	100.0% [19]	
Malaria confirmed					0.5¹
No	86.2% [362]	1.2% [5]	12.6% [53]	100.0% [420]	
Yes	75.0% [3]	0.0% [0]	25.0% [1]	100.0% [4]	
Typhoid fever vaccination					0.2¹
No	86.0% [350]	1.0% [4]	13.0% [53]	100.0% [407]	
Yes	88.2% [15]	5.9% [1]	5.9% [1]	100.0% [17]	
Year					0.005¹
2020	86.7% [340]	0.5% [2]	12.8% [50]	100.0% [392]	
2021	78.1% [25]	9.4% [3]	12.5% [4]	100.0% [32]	
Season					0.5¹
Rainy season	85.7% [222]	0.8% [2]	13.5% [35]	100.0% [259]	

**Characteristics**	**Norovirus not detected** n = 365	**NoVGI detected** n = 5	**NoVGII detected** n = 54	**Total** n = 424	**p-value**
Dry Season	86.7% [143]	1.8% [3]	11.5% [19]	100.0% [165]	
Sample collection center					0.040²
DIELMO	88.6% [31]	0.0% [0]	11.4% [4]	100.0% [35]	
DKR-DISPENSAIRE ST MARTIN	87.5% [7]	12.5% [1]	0.0% [0]	100.0% [8]	
DKR-KEUR JAVOUHEY	95.7% [22]	0.0% [0]	4.3% [1]	100.0% [23]	
GTTE LAIT ST LOUIS	81.7% [49]	0.0% [0]	18.3% [11]	100.0% [60]	
GUED-MEDINA GOUNASS	73.2% [30]	4.9% [2]	22.0% [9]	100.0% [41]	
KAOLACK-ABATTOIRS	100.0% [6]	0.0% [0]	0.0% [0]	100.0% [6]	
KEDOUGOU-DALABA	100.0% [7]	0.0% [0]	0.0% [0]	100.0% [7]	
MATAM-BOKIDIAWE	93.7% [59]	1.6% [1]	4.8% [3]	100.0% [63]	
NDIAYE-NDIAYE-FATICK	100.0% [17]	0.0% [0]	0.0% [0]	100.0% [17]	
NDIOP	73.3% [11]	0.0% [0]	26.7% [4]	100.0% [15]	
PS MBOUR TOUCOULEUR	96.2% [25]	0.0% [0]	3.8% [1]	100.0% [26]	
PS PONT TAMBA	71.7% [33]	0.0% [0]	28.3% [13]	100.0% [46]	
SANTHIABA LOUGA	100.0% [1]	0.0% [0]	0.0% [0]	100.0% [1]	
ST-JOSEPH ZIGUINCHOR	88.0% [66]	1.3% [1]	10.7% [8]	100.0% [75]	
THIES-SERIGNE MOR T.FALL	100.0% [1]	0.0% [0]	0.0% [0]	100.0% [1]	
Region					0.10²
DAKAR	81.9% [59]	4.2% [3]	13.9% [10]	100.0% [72]	
FATICK	88.1% [59]	0.0% [0]	11.9% [8]	100.0% [67]	
KAOLACK	100.0% [6]	0.0% [0]	0.0% [0]	100.0% [6]	
KEDOUGOU	100.0% [7]	0.0% [0]	0.0% [0]	100.0% [7]	
LOUGA	100.0% [1]	0.0% [0]	0.0% [0]	100.0% [1]	
MATAM	93.7% [59]	1.6% [1]	4.8% [3]	100.0% [63]	
SAINT-LOUIS	81.7% [49]	0.0% [0]	18.3% [11]	100.0% [60]	
TAMBA	71.7% [33]	0.0% [0]	28.3% [13]	100.0% [46]	

**Characteristics**	**Norovirus not detected** n = 365	**NoVGI detected** n = 5	**NoVGII detected** n = 54	**Total** n = 424	**p-value**
THIES	96.3% [26]	0.0% [0]	3.7% [1]	100.0% [27]	
ZIGUINCHOR	88.0% [66]	1.3% [1]	10.7% [8]	100.0% [75]	
**Virological results**					
Rota virus					0.3¹
Not Detected	86.0% [345]	1.0% [4]	13.0% [52]	100.0% [401]	
Detected	87.0% [20]	4.3% [1]	8.7% [2]	100.0% [23]	
Adenovirus					0.8¹
Not Detected	85.8% [344]	1.2% [5]	13.0% [52]	100.0% [401]	
Detected	91.3% [21]	0.0% [0]	8.7% [2]	100.0% [23]	
Symptoms					
≥ 3 stools /day					0.7¹
Absence	100.0% [9]	0.0% [0]	0.0% [0]	100.0% [9]	
Presence	85.8% [356]	1.2% [5]	13.0% [54]	100.0% [415]	
Blood in the stool					0.086¹
Absence	86.5% [334]	0.8% [3]	12.7% [49]	100.0% [386]	
Presence	81.6% [31]	5.3% [2]	13.2% [5]	100.0% [38]	
Nausea					0.8¹
Absence	85.8% [350]	1.2% [5]	13.0% [53]	100.0% [408]	
Presence	93.8% [15]	0.0% [0]	6.3% [1]	100.0% [16]	
Headaches					0.080¹
Absence	84.9% [325]	1.3% [5]	13.8% [53]	100.0% [383]	
Presence	97.6% [40]	0.0% [0]	2.4% [1]	100.0% [41]	
Vomiting					0.6¹
Absence	85.3% [279]	1.5% [5]	13.1% [43]	100.0% [327]	

Characteristics	Norovirus not detected n = 365	NoVGI detected n = 5	NoVGII detected n = 54	Total n = 424	p-value
Presence	88.7% [86]	0.0% [0]	11.3% [11]	100.0% [97]	
Arthralgia					>0.9¹
Absence	86.0% [361]	1.2% [5]	12.9% [54]	100.0% [420]	
Presence	100.0% [4]	0.0% [0]	0.0% [0]	100.0% [4]	
Fatigue (Asthenia)					>0.9¹
Absence	85.9% [353]	1.2% [5]	12.9% [53]	100.0% [411]	
Presence	92.3% [12]	0.0% [0]	7.7% [1]	100.0% [13]	
Headaches (Myalgia)					0.4¹
Absence	86.2% [363]	1.2% [5]	12.6% [53]	100.0% [421]	
Presence	66.7% [2]	0.0% [0]	33.3% [1]	100.0% [3]	
Chills					0.5¹
Absence	86.2% [361]	1.2% [5]	12.6% [53]	100.0% [419]	
Presence	80.0% [4]	0.0% [0]	20.0% [1]	100.0% [5]	
Sore throat					>0.9¹
Absence	86.1% [364]	1.2% [5]	12.8% [54]	100.0% [423]	
Presence	100.0% [1]	0.0% [0]	0.0% [0]	100.0% [1]	
Rhinorrhea					0.12¹
Absence	86.9% [339]	1.0% [4]	12.1% [47]	100.0% [390]	
Presence	76.5% [26]	2.9% [1]	20.6% [7]	100.0% [34]	
Cough					0.5¹
Absence	86.2% [293]	0.9% [3]	12.9% [44]	100.0% [340]	
Presence	85.7% [72]	2.4% [2]	11.9% [10]	100.0% [84]	
Abdominal pain					0.7¹

**Characteristics**	**Norovirus not detected** n = 365	**NoVGI detected** n = 5	**NoVGII detected** n = 54	**Total** n = 424	**p-value**
Absence	86.2% [319]	1.1% [4]	12.7% [47]	100.0% [370]	
Presence	85.2% [46]	1.9% [1]	13.0% [7]	100.0% [54]	
Dehydration					>0.9¹
Absence	86.0% [363]	1.2% [5]	12.8% [54]	100.0% [422]	
Presence	100.0% [2]	0.0% [0]	0.0% [0]	100.0% [2]	
Neurological signs					
Absence	86.1% [365]	1.2% [5]	12.7% [54]	100.0% [424]	
Anemia					>0.9¹
Absence	86.0% [363]	1.2% [5]	12.8% [54]	100.0% [422]	
Presence	100.0% [2]	0.0% [0]	0.0% [0]	100.0% [2]	
Fever					0.8¹
Absence	85.8% [343]	1.3% [5]	13.0% [52]	100.0% [400]	
Presence	91.7% [22]	0.0% [0]	8.3% [2]	100.0% [24]	
Other signs					0.8¹
Absence	86.1% [341]	1.3% [5]	12.6% [50]	100.0% [396]	
Presence	85.7% [24]	0.0% [0]	14.3% [4]	100.0% [28]	

Note. NoVG1=Norovirus GI, NoVG2=Norovirus G2

[1] Fisher exact test

[2]Chi-squared test with simulated p-value, (based on 2000 replicates)

Table S2: Data from the regression models applied on clinical data between 2020 and 2021.

**Target variables**	**Models used**	**Significantly associated variables**	**Model quality and discriminatory power**
Study population	Multinomial regression	NoVGI: Year of collection, rotavirus (16 times higher risk), rhinorrhea (2 times higher risk), blood in stool (7 times higher risk), area of residence (urban area slightly lower risk)<br>NoVGII: Year of collection, rotavirus (slightly lower risk), area of residence (urban environment slightly lower risk), myalgia (3 times higher risk)	• R^2Mcfadden =0.052<br>• pseudo-R^2 de Cox & Snell=0.045<br>• AIC=384
NoVGI and NoVGII infections	Binary logistic regression	Area of residence (lower-risk urban area)<br>Year of collection	• AUC= 0.68, R^2Mcfadden =0.084<br>• pseudo-R^2 de Cox & Snell=0.118<br>• AIC=333
Adenovirus infection	Binary logistic regression	Year of collection<br>Headache (12 times more likely)	• AUC= 0.782, R^2Mcfadden =0.16<br>• pseudo-R^2 de Cox & Snell=0.065<br>• AIC=184

Rotavirus infection	Binary logistic regression	Residential area (urban area, slightly less at risk)	• AUC= 0.859, R^2Mcfadden =0.267<br>• pseudo-R^2 de Cox & Snell=0.065<br>• AIC=149.04

NoVGI=Norovirus GI, NoVGII= Norovirus GII, AIC= Akaike Information Criterion, AUC = Area Under the Curve

Table S3: Modelling of NoVGII viral loading wastewater between 2020 and 2023.

Variable	Coefficient (β)	p-value	% Variation	Interpretation
**(Reference)**	–	–	–	Intercept = September, Korentas site, rainy season
**Dry season**	0.749	0.0739	+111.48%	No significant difference related to the season
**Wastewater treatment plants**				
Camberene	3,302	1.67e-07	+2,616.7%	**26 times higher** than in KORENTAS (**significant**)
Canal de pompage UCAD	2,290	0.0002	+887.4%	** Almost 9 times higher **, significant
Canal de Soumbédioune	2,447	8.84e-05	+1,055.4%	10.5 times higher , significant
Cap des Biches	3,589	1.17e-08	+3,519.7%	**35 times higher ** , very significant
Gouye	2,261	0.0009	+859.3%	8.6 times higher , significant
Keur Saib Ndoye	3,288	0.0007	+2,578.7%	** 25.8 times higher **, significant
Khourounar	4,026	1.26e-10	+5,503.0%	**55 times higher ** , very significant
Ouakam	2,924	3.01e-06	+1,761.6%	17.6 times higher , significant
SHS Cité Enseignant	3.865	2.46e-09	+4670.3%	46 times higher , very significant
SP5 Keur Sheikh	2,565	0.0002	+1,200.0%	12 times higher , significant
Station de pompage SOR	2,770	2.04e-05	+1,495.8%	14.9 times higher , significant
Station Keur Niang	1,243	0.126	+246.8%	2.4 times higher (**not significant **)

Station Pompage 1 Kaolack	1.621	0.011	+405.8%	4 times higher , ** significant **
Tivaouane Peulh	3,972	4.32e-10	+5,209.0%	52 times higher , very significant
**Collection month**				
August	0.803	0.050	+123.2%	1.2 times higher that September , deadline significant
April	-0.508	0.332	-39.8%	Decrease (not significant)
December	0.511	0.249	+66.7%	1.5 times higher (not significant)
February	-0.118	0.786	-11.1%	Lightweight decrease , not significant
January	0.643	0.171	+90.2%	0.9 times higher , not significant
July	-0.238	0.548	-26.8%	Drop moderate , not significant
June	0.068	0.884	+7.0%	None notable difference , but not significant
May	0.466	0.312	+59.4%	Lightweight increase , not significant
November	-0.099	0.833	-9.4%	None 10notable difference
October	-0.683	0.086	-49.6%	decrease , close to the significance threshold

Table S4: Modelling of the NoVGI viral load in wastewater between 2020 and 2023.

Modality	Estimate (β)	p-value	Variation (%)	Interpretation
References	19.7944	—	—	Month = January Site= Kaolack Pumping Station Season = season dried
Rainy season	-0.6770	0.288	-49.5%	Viral load reduced by half (not significant)
Wastewater treatment plants				
KORENTAS	-1.7833	0.217	-83.2%	Viral load significantly reduced (not significant)
Camberene	2.0866	0.027	+705.7%	Viral load ~7 times higher (significant)
Canal de pompage UCAD	1.4486	0.125	+325.7%	Viral load ~3 times higher (not significant)
Canal de Soumbédioune	1.3111	0.159	+271.0%	Viral load ~2.7 times higher (not significant)
CAP DES BICHES	0.7322	0.423	+107.9%	Tendency to be ~2 times higher (not significant)
GOUYE	0.6084	0.656	+84.7%	No noticeable difference
KEUR SAIB NDOYE	1.2309	0.383	+242.4%	Tendency to be ~2.4 times higher (not significant)
Khourounar	1.6476	0.068	+419.4%	Tendency to be ~4 times higher (margin of significance)
OUAKAM	1.8818	0.042	+556.5%	Viral load ~5.6 times higher (significant)

SHS Cite Enseignant	1.9536	0.030	+606.4%	Viral load ~6 times higher (significant)
SP5 KEUR CKEIKH	2.1837	0.127	+787.9%	Viral load ~8 times higher (not significant)
Station de pompage Sor	1.4825	0.108	+340.3%	Viral load ~3.4 times higher (not significant)
Station keur Ninag	0.6701	0.580	+95.2%	Tendency to be ~1.04 times higher (not significant)
TIVAOUANE PEULH	1.8788	0.044	+554.6%	Viral load ~5.5 times higher (significant)
Collection month				
March	0.4076	0.598	+50.3%	Tendency to be ~1.9 times higher (not significant)
February	-1.3737	0.018	-74.6%	Viral load ~1.3 times lower (significant)
April	-1.5429	0.062	-78.6%	No noticeable difference
May	-0.9992	0.178	-63.2%	Tendency to be ~1.5 times lower (not significant)
June	-0.6954	0.270	-50%	Tendency to be ~2 times lower (not significant)
July	-0.5139	0.392	-40.2%	Tendency to be ~2 times lower (not significant)
August	-1.0048	0.096	-63.4%	No noticeable difference
September	-0.2509	0.688	-22.2%	Tendency to be ~4 times lower (not significant)
November	-2.6581	0.0001	-93.0%	No noticeable difference

December	0.1945	0.823	+21.4%	Tendency to be ~4 times lower (not significant)

B. Supplementary Figures

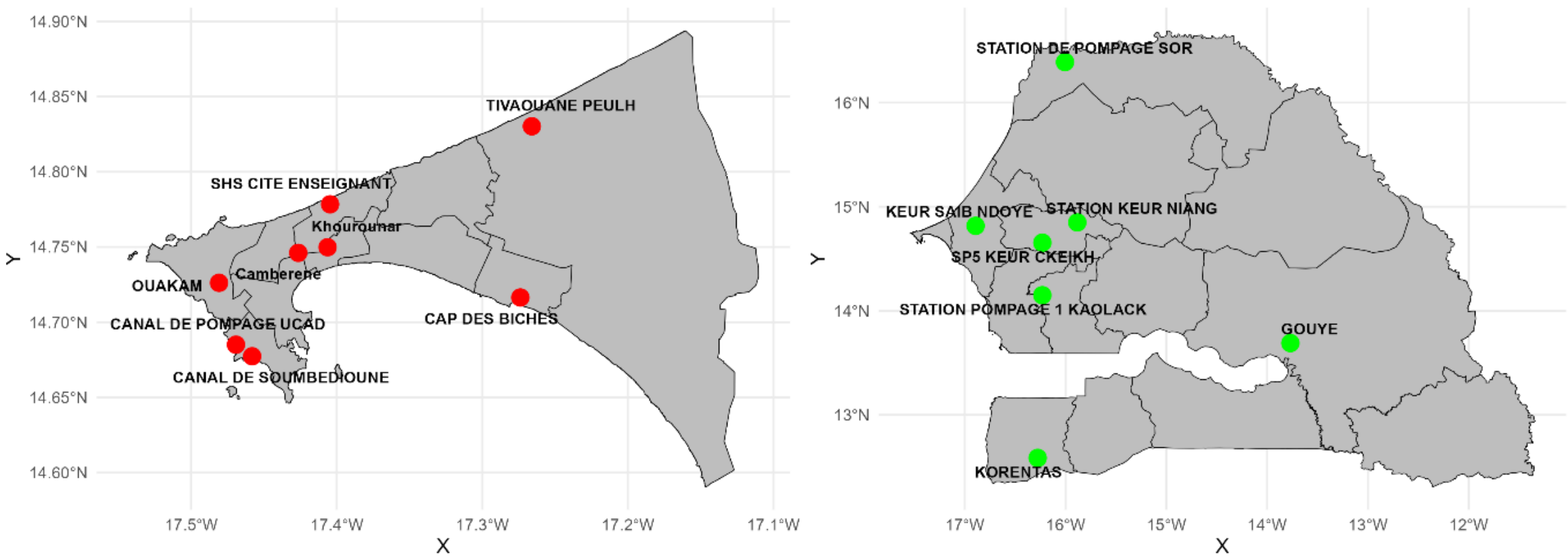

Figure S1: Geographical distribution of wastewater treatment plants. The map locates the different wastewater treatment plants included in the study. The green dots represent stations located in Senegal (outside the Dakar region), while the red dots correspond to stations located in the Dakar region.

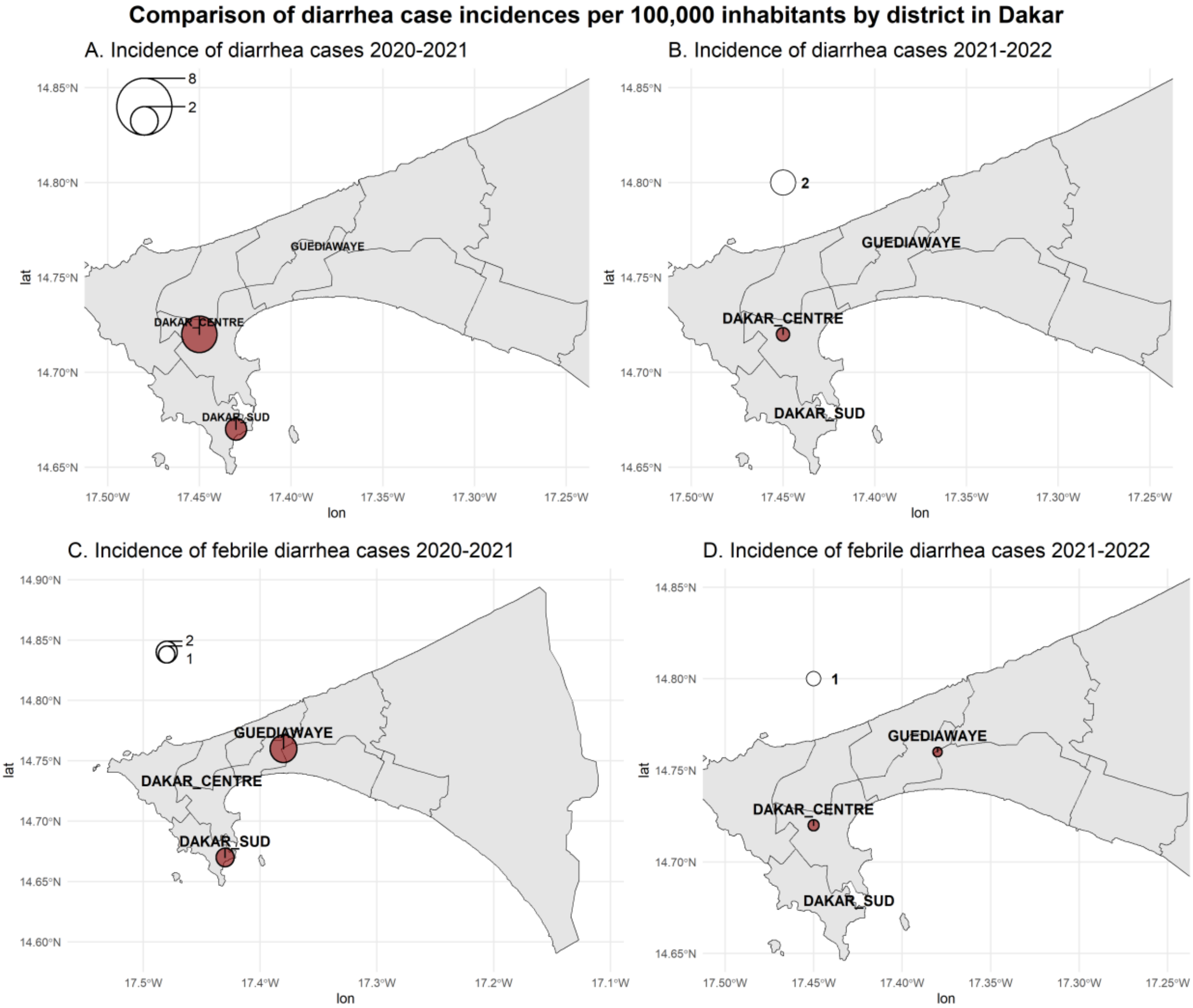

Figure S2: Maps of the incidence of reported cases of diarrhea in clinical data for districts in the Dakar region between 2020 and 2023. The annual incidence is represented by circles in red positioned by district. The size of the circles is proportional to the observed incidence. The nested legend indicates the scale of values.

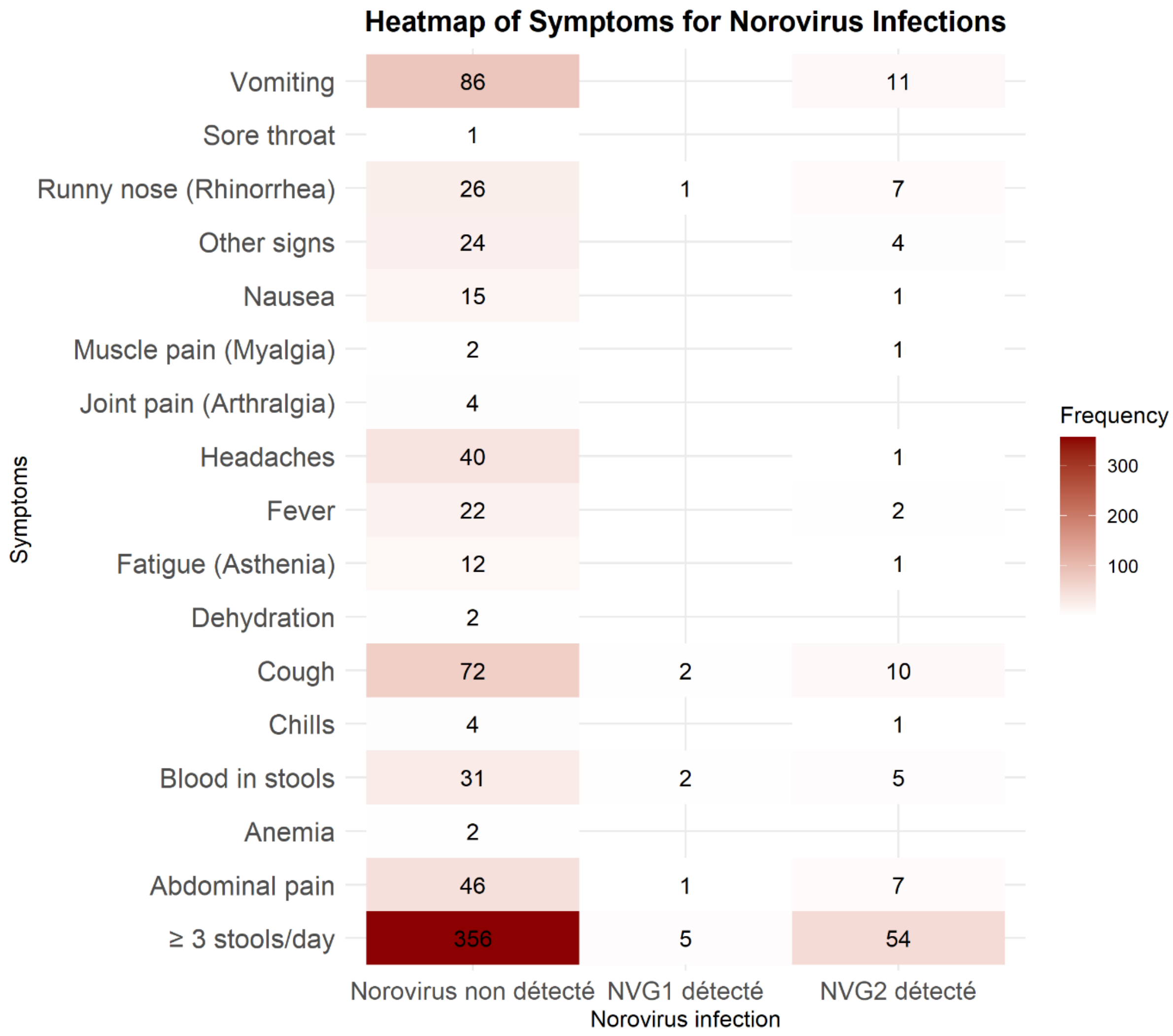

Figure S3: Heat map of symptoms associated with norovirus infections in clinical samples collected between 2020 and 2021. The intensity of the color reflects the frequency of symptoms according to the category of individuals studied: the darker the color, the more frequent the symptom.

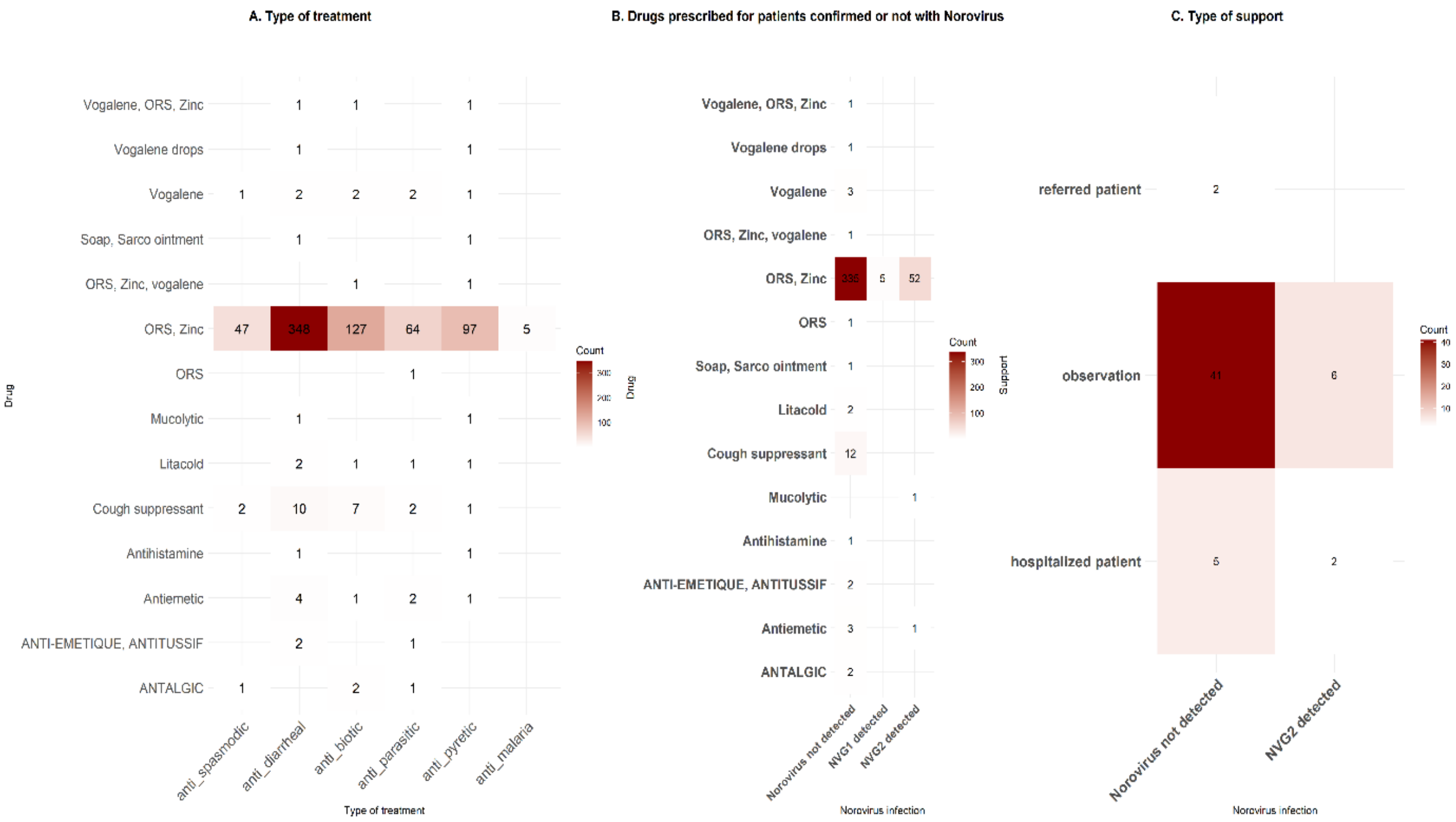

Figure S4: Management and treatment administered to patients with diarrhea reported between 2020 and 2021, depending on whether or not norovirus is detected. A) Frequency of medications administered by type. B) Medications administered according to norovirus infection status. C) Type of care according to the category of individuals studied. The intensity of the color reflects the observed frequency.

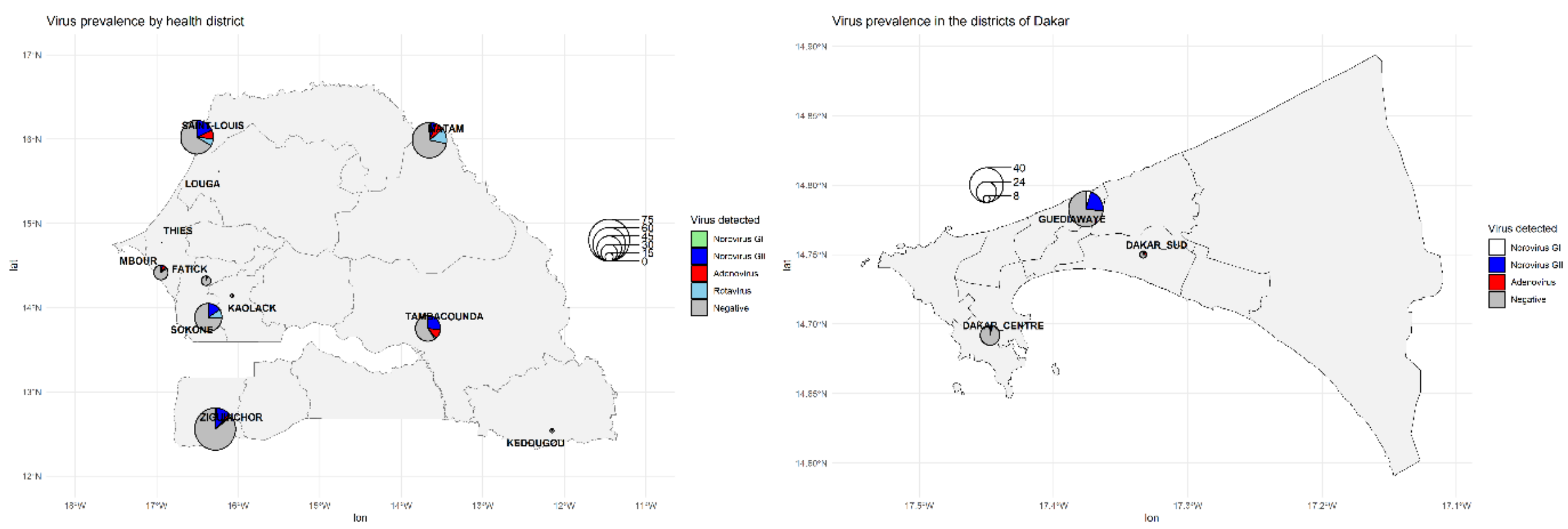

Figure S5: Distribution of the prevalence of the main viruses responsible for gastroenteritis in clinical samples between 2020 and 2021. Prevalence is calculated from confirmed cases and represented by circles positioned by district. The size of the circles is proportional to the number of samples tested. The nested legend indicates the scale of values. The colors indicate: Green: NoVGI prevalence, Dark blue: NoVGII prevalence, Red: Adenovirus prevalence, Light blue: Rotavirus prevalence, Gray: Negative sample rate. In the panel for Dakar, the colors are identical except for NoVGI, which is shown in white.

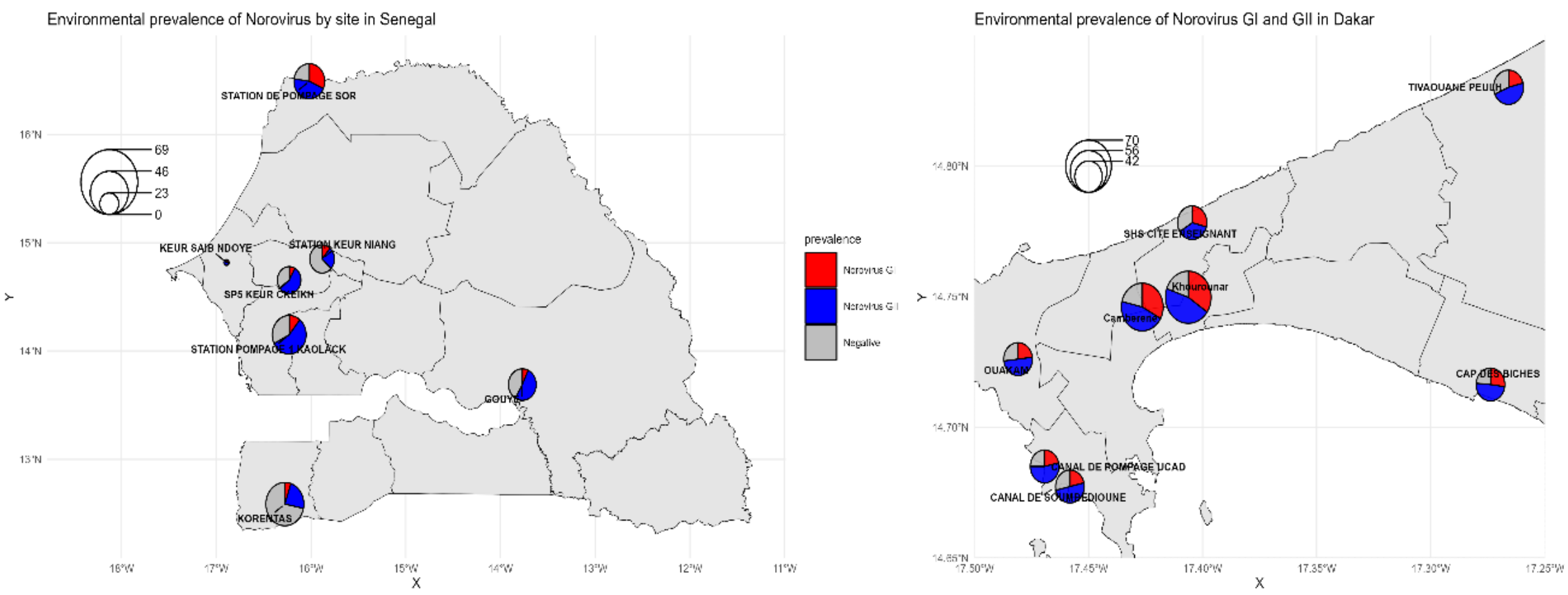

Figure S6: Spatial distribution of the prevalence of norovirus in environmental samples between 2020 and 2023. Prevalence is estimated from wastewater samples and represented by circles positioned by treatment plant. The size of the circles is proportional to the number of samples analyzed. The nested legend indicates the scale of values. The colors correspond to: red: prevalence of NoVGI, blue: prevalence of NoVGII, gray: rate of negative samples.

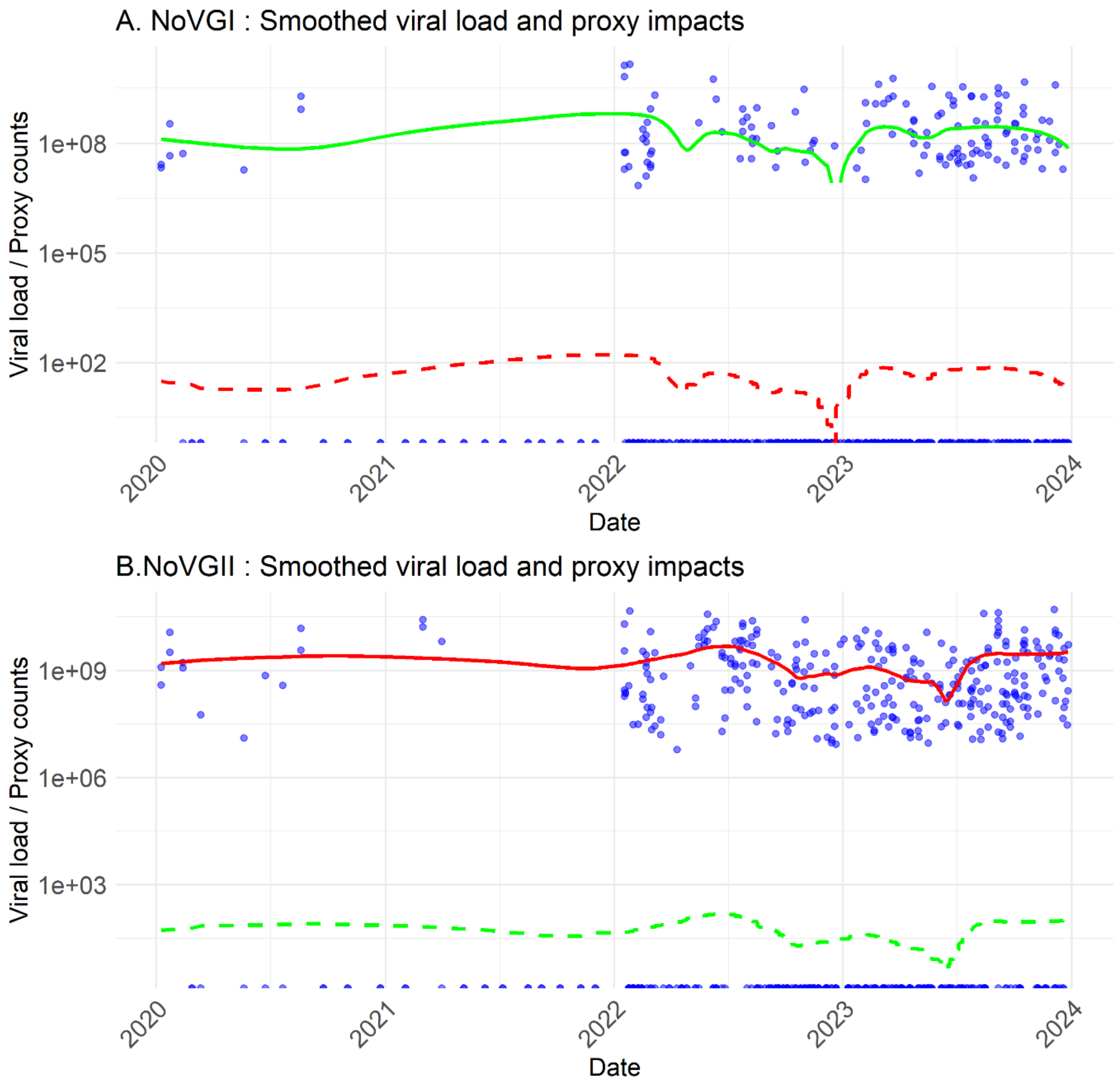

Figure S7: Incidence trends of wastewater using LOESS smoothing and proxy. A) The green curve represents the LOESS smoothing applied to the environmental data, while the blue curve corresponds to the estimated indirect impact indicator. B) The red curve represents the LOESS smoothing applied to the environmental data, while the green curve corresponds to the estimated indirect impact indicator.

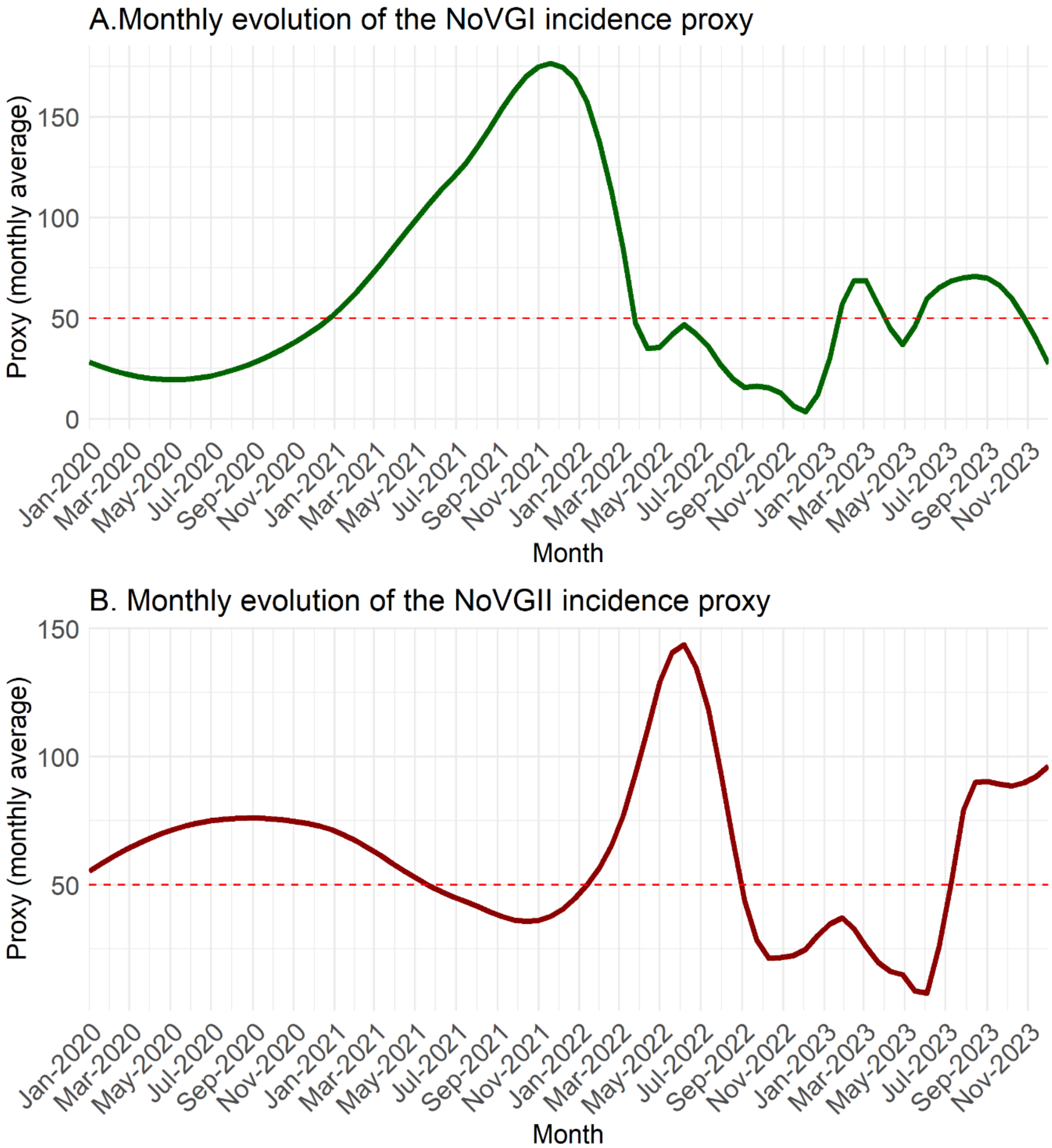

Figure S8: Temporal distribution of the proxy incidence of norovirus in wastewater between 2020 and 2023. The green curve represents the evolution of the NoVGI incidence proxy and the red curve that of NoVGII. The red dotted line indicates the epidemic threshold.

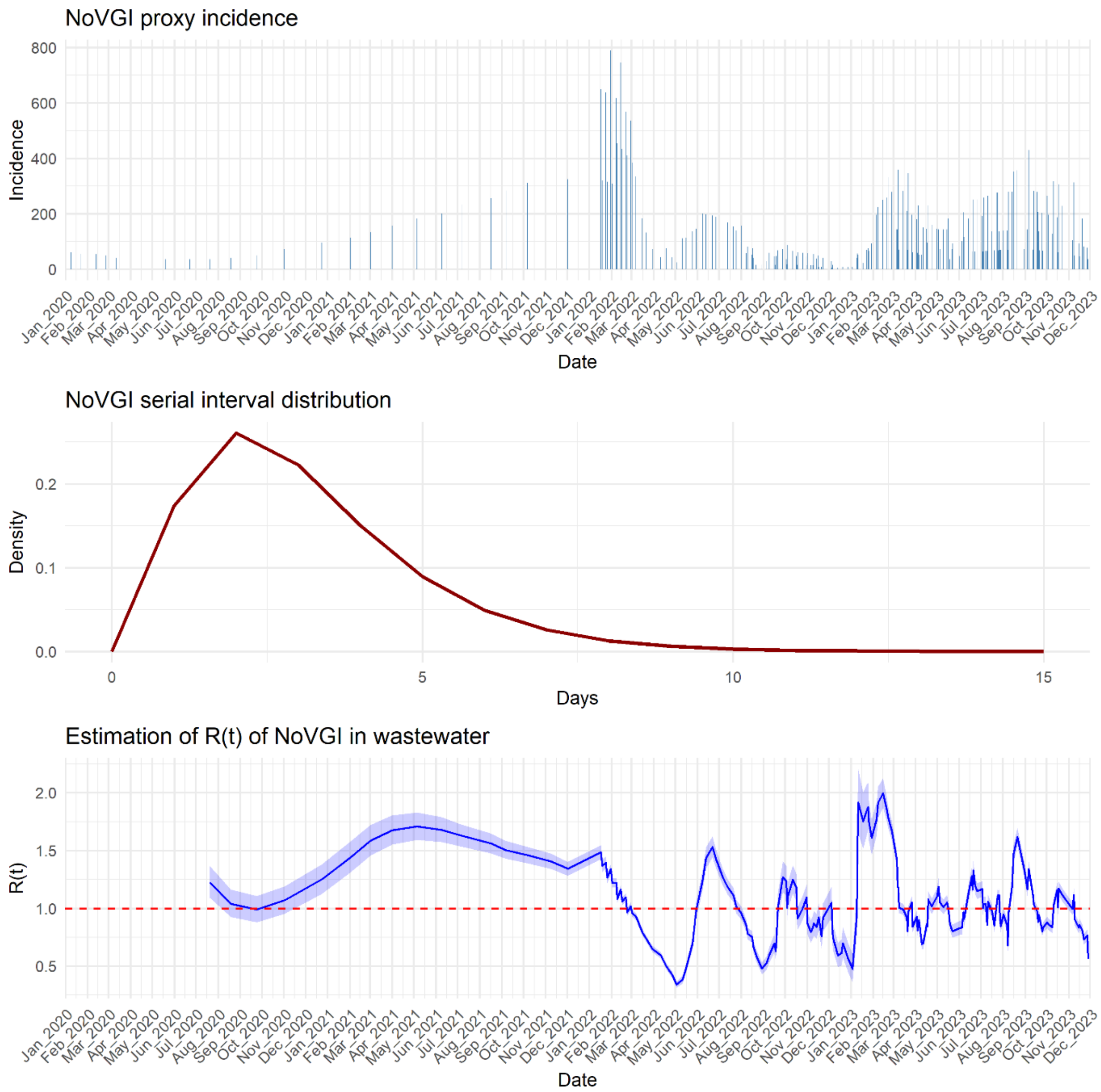

Figure S9: Data of the estimation of NoVGI transmission in wastewater between 2020 and 2023. There are three presentations: the incidence proxy estimate, which tracks the temporal evolution of cases detected in wastewater; the serial interval estimate, corresponding to the average time between a primary infection and the resulting secondary cases; and the reproduction number estimate over time. The red line indicates the epidemic threshold (R = 1).

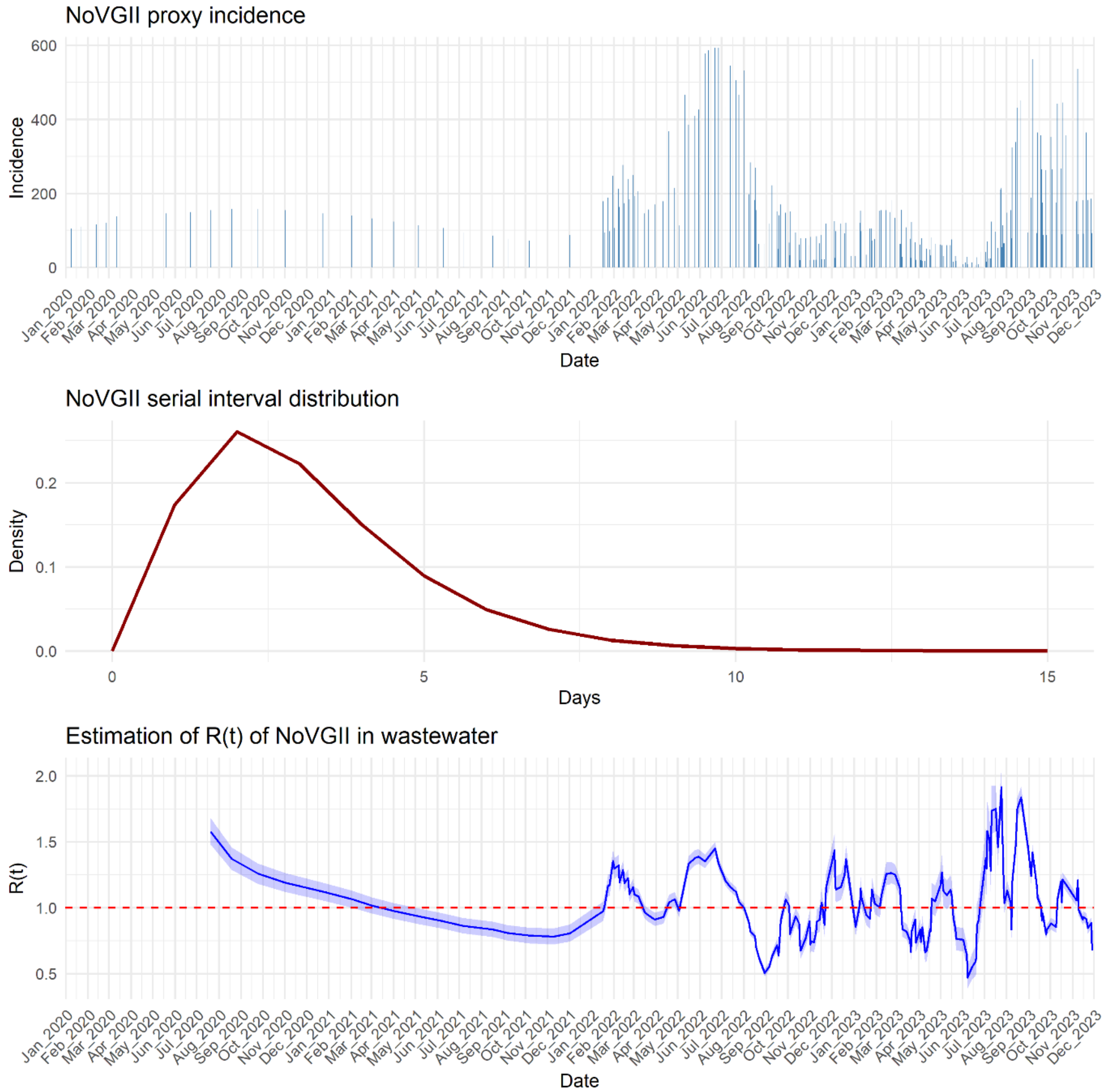

Figure S10: Data of the estimation of NoVGII transmission in wastewater between 2020 and 2023. The figure presents the incidence proxy estimate, serial interval and reproduction number over time for NoVGII. The red line indicates the epidemic threshold (R = 1).
